# Meningococcal Vaccination: Recommendations of the Advisory Committee on Immunization Practices, United States, 2020

**DOI:** 10.15585/mmwr.rr6909a1

**Published:** 2020-09-25

**Authors:** Sarah A. Mbaeyi, Catherine H. Bozio, Jonathan Duffy, Lorry G. Rubin, Susan Hariri, David S. Stephens, Jessica R. MacNeil

**Affiliations:** ^1^Division of Bacterial Diseases, National Center for Immunization and Respiratory Diseases, CDC; ^2^Immunization Safety Office, National Center for Emerging and Zoonotic Infectious Diseases, CDC; ^3^Steven and Alexandra Cohen Children’s Medical Center of New York, New Hyde Park, New York, and Hofstra North Shore–LIJ School of Medicine, Hempstead, New York; ^4^Emory University School of Medicine, Atlanta, Georgia; ^5^Office of the Director, National Center for Immunization and Respiratory Diseases, CDC

## Abstract

This report compiles and summarizes all recommendations from CDC’s Advisory Committee on Immunization Practices (ACIP) for use of meningococcal vaccines in the United States. As a comprehensive summary and update of previously published recommendations, it replaces all previously published reports and policy notes. This report also contains new recommendations for administration of booster doses of serogroup B meningococcal (MenB) vaccine for persons at increased risk for serogroup B meningococcal disease. These guidelines will be updated as needed on the basis of availability of new data or licensure of new meningococcal vaccines.

ACIP recommends routine vaccination with a quadrivalent meningococcal conjugate vaccine (MenACWY) for adolescents aged 11 or 12 years, with a booster dose at age 16 years. ACIP also recommends routine vaccination with MenACWY for persons aged ≥2 months at increased risk for meningococcal disease caused by serogroups A, C, W, or Y, including persons who have persistent complement component deficiencies; persons receiving a complement inhibitor (e.g., eculizumab [Soliris] or ravulizumab [Ultomiris]); persons who have anatomic or functional asplenia; persons with human immunodeficiency virus infection; microbiologists routinely exposed to isolates of *Neisseria meningitidis;* persons identified to be at increased risk because of a meningococcal disease outbreak caused by serogroups A, C, W, or Y; persons who travel to or live in areas in which meningococcal disease is hyperendemic or epidemic; unvaccinated or incompletely vaccinated first-year college students living in residence halls; and military recruits. ACIP recommends MenACWY booster doses for previously vaccinated persons who become or remain at increased risk.

In addition, ACIP recommends routine use of MenB vaccine series among persons aged ≥10 years who are at increased risk for serogroup B meningococcal disease, including persons who have persistent complement component deficiencies; persons receiving a complement inhibitor; persons who have anatomic or functional asplenia; microbiologists who are routinely exposed to isolates of *N. meningitidis;* and persons identified to be at increased risk because of a meningococcal disease outbreak caused by serogroup B. ACIP recommends MenB booster doses for previously vaccinated persons who become or remain at increased risk. In addition, ACIP recommends a MenB series for adolescents and young adults aged 16–23 years on the basis of shared clinical decision-making to provide short-term protection against disease caused by most strains of serogroup B *N. meningitidis*.

## Introduction

Meningococcal disease is a serious bacterial infection that primarily presents as meningitis, bacteremia, or both. Three quadrivalent (serogroups A, C, W, and Y) meningococcal conjugate (MenACWY) vaccines and two serogroup B meningococcal (MenB) vaccines are licensed and available in the United States and are recommended by CDC’s Advisory Committee on Immunization Practices (ACIP) for the prevention of meningococcal disease caused by these serogroups (Table 1) (Box 1) (*1*–*13*). Details about groups recommended to receive meningococcal vaccination, number of vaccine doses, dosing regimens, contraindications, precautions, and special circumstances are described elsewhere in this report.

**TABLE 1:** Licensed and available* meningococcal vaccines — United States, 2020

Vaccine product	Manufacturer	Trade name	Age group	Year licensed
**Conjugate (serogroups A, C, W, and Y)**				
**MenACWY-D**	Sanofi Pasteur	Menactra^†^	9 mos–55 yrs	2005
**MenACWY-CRM**	GlaxoSmithKline	Menveo^§^	2 mos–55 yrs	2010
**MenACWY-TT**	Sanofi Pasteur	MenQuadfi^¶^	≥2 yrs	2020
**Protein based (directed at serogroup B)**				
**MenB-FHbp**	Pfizer	Trumenba**	10–25 yrs	2014
**MenB-4C**	GlaxoSmithKline	Bexsero^††^	10–25 yrs	2015

**Abbreviations:** MenACWY-CRM = meningococcal groups A, C, W, and Y oligosaccharide diphtheria CRM_197_ conjugate vaccine; MenACWY-D = meningococcal groups A, C, W, and Y polysaccharide diphtheria toxoid conjugate vaccine; MenACWY-TT = meningococcal groups A, C, W, and Y polysaccharide tetanus toxoid conjugate vaccine; MenB-4C = four-component meningococcal group B vaccine; MenB-FHbp = meningococcal group B factor H binding protein vaccine.

* Two licensed meningococcal vaccines are no longer available in the United States (Menomune – A/C/Y/W-135 [**Source:** Menomune – A/C/Y/W-135. Package insert. Swiftwater, PA: Sanofi Pasteur; 2016. https://www.fda.gov/media/83562/download] and MenHibrix [**Source:** MenHibRix. Package insert. Rixensart, Belgium: GlaxoSmithKline Biologicals; 2012. https://www.fda.gov/media/83688/download]).

^†^ **Source:** Menactra. Package insert. Swiftwater, PA: Sanofi Pasteur; 2018. https://www.fda.gov/media/75619/download

^§^ **Source:** Menveo. Package insert. Sovicille, Italy: GlaxoSmithKline Vaccines; 2019. https://www.fda.gov/media/78514/download

^¶^ **Source:** MenQuadfi. Package insert Swiftwater, PA: Sanofi Pasteur; 2020. https://www.fda.gov/media/137306/download

** **Source:** Trumenba. Package insert. Philadelphia, PA: Pfizer; 2018. https://www.fda.gov/media/89936/download

^††^ **Source:** Bexsero. Package insert. Sovicille, Italy: GlaxoSmithKline Vaccines; 2018. https://www.fda.gov/media/90996/download

BOX 1 **Meningococcal vaccination recommendations — Advisory Committee on Immunization Practices, United States, 2020**

**ACIP recommends MenACWY vaccination for the following groups:**

- Routine vaccination for adolescents aged 11 or 12 years, with a booster dose at age 16 years.
- Routine vaccination of persons aged ≥2 months at increased risk for meningococcal disease (dosing schedule varies by age and indication, and interval for booster dose varies by age at time of previous vaccination):
  ° Persons with certain medical conditions including anatomic or functional asplenia, complement component deficiencies (e.g., C3, C5-C9, properdin, factor H, or factor D), complement inhibitor (e.g., eculizumab [Soliris] or ravulizumab [Ultomiris]) use, or human immunodeficiency virus infection.
  ° Microbiologists with routine exposure to *Neisseria meningitidis* isolates.
  ° Persons at increased risk during an outbreak (e.g., in community or organizational settings, and among men who have sex with men [MSM]).
  ° Persons who travel to or live in countries in which meningococcal disease is hyperendemic or epidemic.
  ° Unvaccinated or undervaccinated first-year college students living in residence halls.
  ° Military recruits.
- Booster doses for previously vaccinated persons who become or remain at increased risk.

**ACIP recommends MenB vaccination for the following groups:**

- Routine vaccination of persons aged ≥10 years at increased risk for meningococcal disease (dosing schedule varies by vaccine brand; boosters should be administered at 1 year after primary series completion, then every 2–3 years thereafter):
  ° Persons with certain medical conditions, such as anatomic or functional asplenia, complement component deficiencies, or complement inhibitor use.
  ° Microbiologists with routine exposure to *N. meningitidis* isolates.
  ° Persons at increased risk during an outbreak (e.g., in community or organizational settings, and among MSM).
- Vaccination of adolescents and young adults aged 16–23 years with a 2-dose MenB series on the basis of shared clinical decision-making. The preferred age for MenB vaccination is 16–18 years. Booster doses are not recommended unless the person becomes at increased risk for meningococcal disease.
- Booster doses for previously vaccinated persons who become or remain at increased risk.

**Abbreviations:** ACIP = Advisory Committee on Immunization Practices; MenACWY = quadrivalent (serogroups A, C, W, Y) meningococcal conjugate vaccine; MenB = serogroup B meningococcal vaccine.

This report compiles and summarizes all previously published ACIP recommendations for use of meningococcal vaccines in the United States (Box 2) (*1*–*15*). It also clarifies certain existing recommendations and contains new recommendations for administration of booster doses of MenB vaccine among persons aged ≥10 years at increased risk for serogroup B meningococcal disease. This report is intended for use by clinicians and public health providers for guidance regarding the use of meningococcal vaccines.

BOX 2 **Timeline of meningococcal vaccine licensure and recommendations, United States, 2005—2020**

2005 FDA licensed MenACWY-D for persons aged 11–55 years. ACIP recommended routine vaccination of adolescents with a single MenACWY-D dose at age 11–12 years and persons aged 11–55 years at increased risk for meningococcal disease.

2006 Because of limited vaccine supply, MenACWY-D vaccination was limited to cohorts of adolescents entering high school and college and persons aged 11–55 years at increased risk for meningococcal disease.

2007 After vaccine supply became sufficient, ACIP recommended vaccination for all adolescents aged 11–18 years. FDA expanded licensure of MenACWY-D to children aged 2–10 years, and ACIP recommended routine vaccination of children in this age group at increased risk for meningococcal disease.

2010 FDA licensed a second vaccine, MenACWY-CRM, for persons aged 11–55 years. ACIP added a MenACWY booster dose at age 16 years and recommended a 2-dose primary series be used for certain persons aged 11–55 years at increased risk for meningococcal disease because of asplenia, persistent complement component deficiency, or human immunodeficiency virus (HIV) infection (with another indication for vaccination).

2011 FDA extended licensure of MenACWY-CRM to children aged 2–10 years and of MenACWY-D to those aged 9–23 months. ACIP recommended a 2-dose primary series of MenACWY-D for children aged 9–23 months at increased risk for meningococcal disease.

2012 FDA licensed Hib-MenCY-TT and ACIP recommended a 4-dose primary series for children aged 2–8 months at increased risk for meningococcal disease.

2013 FDA extended licensure of MenACWY-CRM to children aged 2–23 months and ACIP recommended a 4-dose primary series for children in this age group at increased risk for meningococcal disease.

2014 FDA licensed MenB-FHbp as a 3-dose series for persons aged 10–25 years.

2015 FDA licensed MenB-4C as a 2-dose series for persons aged 10–25 years. ACIP recommended persons at increased risk for serogroup B meningococcal disease receive a MenB series, and persons aged 16–23 years were recommended to be vaccinated with a MenB series on the basis of shared clinical decision-making.

2016 FDA licensed MenB-FHbp as a 2-dose series for persons aged 10–25 years.

2016 ACIP recommended persons with HIV infection be routinely vaccinated with a 2-dose MenACWY primary series.

2017 ACIP updated its recommendations for use of MenB-FHbp following a change in licensure that allowed both a 2- and 3-dose series. Distribution of MPSV4 and Hib-MenCY-TT was discontinued in the United States.

2019 ACIP recommended that persons with certain medical conditions and microbiologists routinely exposed to *Neisseria meningitidis* isolates receive a MenB booster dose 1 year after primary series completion, then every 2–3 years thereafter. During an outbreak, a single MenB booster dose was recommended if it had been ≥1 year since primary series completion (interval of ≥6 months may be considered if recommended by public health officials).

2020 FDA licensed MenACWY-TT for persons aged ≥2 years.

**Abbreviations:** ACIP = Advisory Committee on Immunization Practices; FDA = Food and Drug Administration; Hib-MenCY-TT = meningococcal groups C and Y and *Haemophilus* *influenzae* type b tetanus toxoid vaccine (MenHibrix); MenACWY-CRM = meningococcal groups A, C, W, and Y oligosaccharide diphtheria CRM_197_ conjugate vaccine (Menveo); MenACWY-D = meningococcal groups A, C, W, and Y polysaccharide diphtheria toxoid conjugate vaccine (Menactra); MenACWY-TT = meningococcal groups A, C, W, and Y polysaccharide tetanus toxoid conjugate vaccine (MenQuadfi); MenB = serogroup B meningococcal vaccine; MenB-4C = meningococcal group B vaccine (Bexsero); MenB-FHbp = meningococcal group B vaccine (Trumenba); MPSV4 = meningococcal polysaccharide vaccine, groups A, C, Y, and W combined (Menomune – A/C/Y/W-135).

## Methods

ACIP provides recommendations for the prevention and control of meningococcal disease in the United States. The ACIP Meningococcal Vaccines Work Group met by teleconference once per month during 2005–2020, except during brief periods of hiatus. Work group membership included voting members of ACIP, representatives of ACIP ex-officio and liaison organizations, and scientific consultants with expertise in public health, vaccinology, medical specialties, vaccine research, and assessments of vaccine efficacy and safety. Work group discussions included topics such as meningococcal disease surveillance and epidemiology and meningococcal vaccine safety, immunogenicity, effectiveness, coverage, program feasibility, and cost-effectiveness. Presentations were requested from invited experts, and published and unpublished data were discussed. These data were summarized by the work group and presented to ACIP to help establish recommendations. When evidence was lacking, the recommendations incorporated expert opinion from ACIP. Meeting minutes and information on ACIP membership and conflicts of interest are available on the ACIP website (https://www.cdc.gov/vaccines/acip). This report updates and replaces previously published ACIP recommendations for meningococcal vaccines (*9*–*13*,*16*).

Grading of Recommendations, Assessment, Development and Evaluation (GRADE) was adopted by ACIP in 2010 (*17*). Recommendations using the GRADE approach include the use of MenACWY oligosaccharide diphtheria CRM_197_ conjugate vaccine (MenACWY-CRM) among children aged 2–23 months at increased risk for meningococcal disease, use of a MenACWY vaccine among persons infected with human immunodeficiency virus (HIV), all MenB vaccine recommendations, and use of a MenACWY tetanus toxoid vaccine (MenACWY-TT) among persons aged ≥2 years (*9*–*11*,*13*). GRADE evidence tables for these recommendations are available (https://www.cdc.gov/vaccines/acip/recs/grade/table-refs.html). In 2018, ACIP adopted the Evidence to Recommendations (EtR) framework to facilitate the assessment and ensure transparency of additional factors considered in developing vaccine recommendations, including target population values, stakeholder acceptability, and feasibility of implementation (*18*). Recommendations for MenB booster doses among persons aged ≥10 years at increased risk for meningococcal disease and use of MenACWY-TT among persons aged ≥2 years were further evaluated using the EtR framework (*19*). The EtR frameworks for these recommendations are available (https://www.cdc.gov/vaccines/acip/recs/grade/etr.html). ACIP did not use GRADE or EtR for updates of evidence related to recommendations made before implementation of these approaches.

ACIP votes were held when a new routine or risk-based recommendation was under consideration, with new age indications or dosing regimens for a vaccine, or when additional groups were identified as being at risk for meningococcal disease. An ACIP vote was not required for use of newly licensed products (e.g., MenACWY-TT in persons aged ≥2 years) when no changes in recommendations for vaccine use in terms of dosing and schedules were made.

For this report, a systematic literature search was completed to review all available evidence on the immunogenicity, effectiveness, and safety of U.S.-licensed MenACWY and MenB vaccines among age groups for which the vaccines were approved, including separate reviews to assess evidence related to MenB booster doses and MenACWY-TT. PubMed, Medline, Embase, CINAHL, Scopus, Cochrane Library, and ClinicalTrials.gov were searched for clinical trials or observational studies published during 2000–2018 without language or geographic restrictions. The following search terms were used: “(quadrivalent meningococcal conjugate or tetravalent meningococcal conjugate or meningococcal ACWY or MCV4 or MCV-4 or MenACWY or MenACWY-D or MenACWY-CRM or Menactra or Menveo or serogroup B meningococcal or meningococcal serogroup B or meningococcal B or meningococcal group B or group B meningococcal or MenB or Bexsero or MenB-4C or rMenB+OMV NZ or 4CMenB or Trumenba or rLP2086 or MenB-FHbp or FHbp or Factor H binding protein)” and “vaccin*” and “(immunogenicity or efficacy or effectiveness or impact or safety or adverse event*).” For the review specific to MenB booster vaccination, the same MenB vaccine search terms plus the term “booster” were used to capture all studies related to booster doses. To identify evidence related to MenACWY-TT, licensed after the original systematic literature search was conducted, as well as newly available evidence related to other meningococcal vaccines, search results were supplemented by data (using updated search terms to include “MenACWY-TT,” “MenACYW-TT,” and “MenQuadfi”) published in 2019–2020 or identified by work group subject matter experts, or unpublished data provided by the vaccine manufacturers.

To further assess vaccine safety, data were evaluated from the Vaccine Adverse Event Reporting System (VAERS) and the Vaccine Safety Datalink (VSD), two postlicensure surveillance systems for adverse events. VAERS is a national passive public health surveillance system operated by CDC and the Food and Drug Administration (FDA) and accepts reports from anyone, including health care professionals, vaccine manufacturers, patients, and caregivers (*20*). Health care providers and patients are encouraged to report clinically important or unexpected adverse events, even if unsure whether the event is vaccine related, and to provide medical documentation for reports of serious adverse events (e.g., death, life-threatening health event, hospitalization, or lasting disability after vaccination) (*20*). VAERS can identify rare adverse events and detect possible safety problems quickly, generating vaccine safety hypotheses to be evaluated by other sources; however, VAERS data cannot be used to determine whether a vaccine caused an adverse event. VSD is a collaboration between CDC and eight health care organizations that conducts active public health surveillance and epidemiologic research about vaccine safety. VSD collects individual-level data, including medical and vaccine records, on approximately 10 million persons annually (approximately 3% of the U.S. population) (*21*). This allows for population-based observational studies with longitudinal follow-up that can be used to calculate rates and relative risks of vaccine adverse events. Therefore, VSD data can be used for surveillance to identify vaccine safety signals or for hypothesis testing to evaluate signals originating from other sources such as VAERS.

## Background

Meningococcal disease includes the spectrum of invasive infections caused by *Neisseria meningitidis*, a gram-negative diplococcus. Meningococcal disease usually presents clinically as meningitis, bacteremia, or both (*22*). Meningococcal disease also can present as other invasive syndromes such as bacteremic pneumonia, arthritis, and pericarditis. Noninvasive infections such as pneumonia without bacteremia, conjunctivitis, or urethritis also might occur. Meningococcal disease develops rapidly, often among previously healthy persons, and results in high morbidity and mortality. Even with appropriate antimicrobial therapy, the overall case-fatality ratio in the United States is 15%, and 10%–20% of survivors have long-term sequelae such as neurologic disability, limb or digit loss, or hearing loss (*22*,*23*).

*N. meningitidis* is classified into 12 serogroups according to the composition of its polysaccharide capsule; serogroups A, B, C, W, X, and Y cause most of the disease globally (*24*). *N. meningitidis* colonizes mucosal surfaces of the nasopharynx and is transmitted through direct contact with large-droplet respiratory tract secretions from patients or asymptomatic carriers. Nasopharyngeal carriage rates are highest in adolescents and young adults, who serve as reservoirs for transmission of *N. meningitidis* (*25*). Invasive disease is an infrequent consequence of nasopharyngeal colonization.

### Epidemiology of Meningococcal Disease in the United States

Since the late 1990s, the incidence of meningococcal disease has steadily decreased in the United States, from 1.2 cases per 100,000 population in 1996 to an historic low of 0.1 cases per 100,000 population in 2018. During 2015–2018, approximately 360 cases occurred annually in the United States, representing an average annual incidence of 0.11 cases per 100,000 population (*26*). Incidence is highest among infants aged <1 year, followed by children aged 1 year and adolescents and young adults aged 16–20 years (*23*). During 2015–2018, the primary serogroups that caused disease were B and C, causing 42% and 26% of cases in which serogroup was known, respectively; serogroups W and Y and nongroupable strains each caused 9%–14% of cases (*26*). Decreases in meningococcal disease incidence began before the introduction of MenACWY and MenB vaccines and have been observed across all age groups and for the predominant disease-causing serogroups in the United States (*23*). Outbreaks account for approximately 5% of meningococcal disease cases across age groups in the United States (*27*). In recent years, several outbreaks of serogroup B meningococcal disease among university students and serogroup C meningococcal disease among men who have sex with men (MSM) have been reported (*28*,*29*).

### Groups at Increased Risk for Meningococcal Disease

Risk factors for meningococcal disease include antecedent viral infection, household crowding, and smoking (*30*–*34*). In addition, certain groups are at increased risk for meningococcal disease, including the following:

- **Persons with persistent complement component deficiencies:** Persons who have persistent (e.g., genetic) deficiencies in the complement pathway (e.g., C3, C5–C9, properdin, factor D, or factor H) have up to a 10,000-fold increased risk for meningococcal disease (*35*). Persons with complement deficiencies might experience recurrent disease and inherited disorders might affect additional family members; therefore, testing for complement deficiency should be considered for patients with meningococcal disease (*36*–*38*).
- **Persons who use complement inhibitors:** Use of complement inhibitors (e.g., the currently licensed eculizumab [Soliris] and its long-acting derivative ravulizumab [Ultomiris] monoclonal antibody therapies that block C5) is associated with a substantially increased risk for meningococcal disease (*39*,*40*). Eculizumab use is associated with an approximately 2,000-fold increased incidence of meningococcal disease (*41*). Complement inhibitor recipients remain at risk for meningococcal disease even after meningococcal vaccination; therefore, CDC guidance indicates that providers could consider treating patients with antimicrobial prophylaxis for the duration of complement inhibitor treatment (*42*).
- **Persons with anatomic or functional asplenia:** Persons with anatomic or functional asplenia (including sickle cell disease) appear to be at increased risk for meningococcal disease and, compared with healthy persons, have a higher mortality rate (40%–70%) from the disease (*43*–*45*).
- **Persons living with HIV infection:** In studies from the United States, United Kingdom, and South Africa, persons living with HIV infection or acquired immunodeficiency syndrome have an elevenfold to twenty-four-fold increased risk for meningococcal disease (*46*–*50*). Among persons living with HIV infection, low CD4 count or high viral load are associated with greater risk (*48*). Most meningococcal cases reported among persons living with HIV infection in the United States are caused by serogroups C, W, or Y (*47*).
- **Microbiologists routinely exposed to *N. meningitidis* isolates:** The annual attack rate of laboratory-acquired meningococcal infection among microbiologists who routinely work with *N. meningitidis* isolates has historically been estimated to be 13 per 100,000 persons, which is manyfold higher than the rate for adults among the general population (*51*). This increased risk is likely related to mechanical manipulation of isolates that generates droplets or aerosols; increased risk was not observed among laboratory workers who handle clinical specimens but not isolates (*51*).
- **Persons at increased risk during an outbreak of meningococcal disease:** Approximately 5% of U.S. cases are outbreak related (*27*). Outbreaks can occur in community or organizational settings (*52*). During outbreaks, the median attack rate is up to 1,400-fold higher than in the nonoutbreak setting (*27*).
- **Travelers to countries where meningococcal disease is hyperendemic or epidemic:** Travelers to countries where meningococcal disease is hyperendemic or epidemic, such as the meningitis belt of sub-Saharan Africa, are at increased risk for exposure, and thus, disease. Historically, serogroup A was the predominant meningococcal pathogen in the meningitis belt. However, after the implementation of a meningococcal serogroup A conjugate vaccine (MenAfriVac), serogroup A disease has been nearly eliminated in the meningitis belt (*53*). Endemic meningococcal disease and outbreaks are now most commonly caused by serogroups C, W, and X (*54*).
- **College students:** Historically, college freshman living in residence halls were identified as being at increased risk for meningococcal disease (*55*). With improved control of serogroups C, W, and Y disease after widespread use of MenACWY vaccine among adolescents, the risk for meningococcal disease among college students is greatest for serogroup B, with a relative risk of 3.5 compared with persons not attending college, although serogroup B disease incidence among this population is low (0.17 cases per 100,000 population) (*56*). Risk factors for serogroup B meningococcal disease among undergraduate college students include age 18–20 years, attendance at a 4-year college, freshman class year, and on-campus residence (*56*,*57*). Although not assessed outside of outbreak settings, participation in a fraternity or sorority is an additional risk factor during serogroup B meningococcal disease outbreaks (*57*).
- **Military recruits:** Historically, new military recruits were identified as being at increased risk for meningococcal disease and outbreaks, most likely related to the crowded living conditions among persons originating from different geographic areas carrying diverse *N. meningitidis* strains (*58*,*59*).
- **Men who have sex with men:** Several outbreaks of serogroup C meningococcal disease have been reported among MSM in the United States (*28*). MSM have also been shown to be at increased risk for meningococcal disease outside of outbreaks, although the incidence of disease remains low. HIV infection might be an important factor for this increased risk in the United States, particularly in nonoutbreak settings (*60*).

### Clinical and Public Health Management of Meningococcal Disease

Early recognition of meningococcal disease is important for prompt diagnosis and initiation of antimicrobial therapy. Symptoms of meningitis include sudden onset of high fever, headache, nuchal rigidity, altered mental status, photophobia, nausea, and vomiting. Meningococcemia might present as fever; malaise; cold hands and feet; leg or other body pain; vomiting; diarrhea; and maculopapular, petechial, or purpuric rash (*61*). The diagnosis of confirmed meningococcal disease is made either through isolation of *N. meningitidis* or detection of *N. meningitidis*–specific nucleic acid in a specimen obtained from a normally sterile body site (e.g., blood or cerebrospinal fluid) (*62*). Culture is the preferred confirmatory test because it allows for further characterization of the strain; however, serogroup-specific polymerase chain reaction testing using a validated assay is a sensitive method for identifying *N. meningitidis*, particularly in situations in which antimicrobial therapy was initiated before specimen collection (*63*). Because *N. meningitidis* infections typically are severe, antibiotics should be started immediately for a patient with suspected meningococcal disease without waiting for laboratory confirmation. Several antibiotics are available for the treatment of meningococcal disease, including ceftriaxone, cefotaxime, and, when the diagnosis is confirmed, penicillin (*64*).

Antimicrobial chemoprophylaxis of close contacts of a patient with meningococcal disease is important to prevent secondary cases. Several antibiotics are recommended for chemoprophylaxis, including ciprofloxacin, rifampin, and ceftriaxone; azithromycin can be used in areas with sustained ciprofloxacin resistance (currently rare in the United States). Additional information on identification of close contacts and administration of chemoprophylaxis is available in CDC’s *Manual for the Surveillance of Vaccine-Preventable Diseases* (*65*).

The investigation of and response to meningococcal disease outbreaks rely on comprehensive epidemiologic and laboratory investigation of cases, identification of persons at increased risk for meningococcal disease as a result of the outbreak and, in certain situations, implementation of vaccination campaigns and use of expanded antimicrobial chemoprophylaxis. Additional information is available in CDC’s *Guidance for the Evaluation and Public Health Management of Suspected Outbreaks of Meningococcal Disease* (*52*).

## Meningococcal Vaccines

Three quadrivalent meningococcal conjugate (MenACWY) vaccines are currently licensed and available in the United States: 1) meningococcal groups A, C, W, and Y polysaccharide diphtheria toxoid conjugate vaccine (MenACWY-D) (Menactra); 2) meningococcal groups A, C, W, and Y oligosaccharide diphtheria CRM_197_ conjugate vaccine (MenACWY-CRM) (Menveo); and 3) meningococcal groups A, C, W, and Y polysaccharide tetanus toxoid conjugate vaccine (MenACWY-TT) (MenQuadfi) (Table 1). Additional information is available in the package inserts (*66*–*68*).

In addition, two serogroup B meningococcal (MenB) vaccines are licensed and available in the United States: 1) MenB-FHbp (Trumenba) and 2) MenB-4C (Bexsero) (Table 1). MenB-FHbp consists of two purified recombinant lipidated FHbp antigens, one from each FHbp subfamily (A and B). MenB-4C consists of three recombinant proteins (neisserial adhesin A [NadA], factor H binding protein [FHbp] fusion protein from subfamily B, and neisserial heparin binding antigen [NhbA] fusion protein) and outer membrane vesicles (OMVs) containing outer membrane protein porin A (PorA) serosubtype P1.4. Additional information on MenB vaccines is available in the package inserts (*69*,*70*).

Two additional licensed meningococcal vaccines are no longer available in the United States: 1) a quadrivalent (serogroups A, C, W, and Y) meningococcal polysaccharide vaccine (MPSV4) (Menomune – A/C/Y/W-135) and 2) a combined *Haemophilus influenzae* type b and meningococcal serogroups C and Y conjugate vaccine (Hib-MenCY-TT) (MenHibrix) (*71*,*72*).

## Evaluation of Efficacy of Meningococcal Vaccines

Because of the low incidence of meningococcal disease in the United States, vaccine efficacy estimates supporting U.S. licensure of the current meningococcal vaccines are based on demonstration of specific immune responses (e.g., immune correlate of protection through serum bactericidal activity [SBA]) and not direct evidence of clinical effectiveness). Protection against invasive meningococcal disease is mediated by bactericidal antibodies to meningococcal capsular polysaccharides or protein antigens in the presence of complement. This complement-dependent bactericidal activity is measured by use of an SBA assay with a human (hSBA) or baby rabbit (rSBA) complement source (*73*). SBA activity has been demonstrated to correlate with immunity against meningococcal disease and thus is accepted as the correlate of protection (*74*). Because meningococci have greater susceptibility to lysis by rabbit complement, antibody titers measured by an rSBA assay are elevated compared with those from an hSBA assay; thus, antibody titers measured by these two assays are not directly comparable (*74*). An hSBA titer ≥1:4 (although a threshold of ≥1:8 also has been used to account for assay variability) or an rSBA titer ≥1:8 and/or a fourfold rise in rSBA or hSBA titers have been used to infer vaccine-mediated immunologic protection against meningococcal disease (*73*).

For the purposes of U.S. licensure, immunogenicity was assessed as the proportion of persons who achieved an SBA titer above a predefined threshold or fourfold rise in SBA titers for serogroups A, C, W, and Y and serogroup B strains tested. For MenACWY vaccines, efficacy was inferred using either rSBA or hSBA. For MenB vaccines, efficacy was inferred using hSBA. MenB vaccines are not expected to protect against all strains of serogroup B *N. meningitidis* because their mechanism of action is against subcapsular proteins and not the polysaccharide capsule (*75*,*76*). Because laboratory evaluation of vaccine efficacy against all serogroup B meningococcal strains would be impossible because of their antigenic and genetic diversity, efficacy of MenB vaccines was inferred using hSBA titers against selected strains (*77*).

Because MenB vaccines do not protect against all strains of serogroup B *N. meningitidis*, assays have been developed to provide additional insight into breadth of strain coverage. For MenB-FHbp, a flow cytometric meningococcal antigen surface expression (MEASURE) assay was developed to quantify the level of FHbp expressed in serogroup B strains through mean fluorescence intensity. On the basis of this assay, 91% of U.S. and European serogroup B strains expressed sufficient FHbp to be susceptible to MenB-FHbp–mediated bactericidal killing (*75*). The meningococcal antigen typing system (MATS) was developed to predict MenB-4C strain coverage using genotyping for PorA and enzyme-linked immunosorbent assays for FHbp, NhbA, and NadA. Using MATS, 91% of U.S. serogroup B meningococcal disease strains are predicted to be covered by MenB-4C, with FHbp and NhbA the greatest contributors to U.S. strain coverage (*76*). A complementary strain coverage prediction method using genotyping (gMATS) also has been developed (*78*), as has the Bexsero antigen sequence type (BAST) scheme to facilitate genomic surveillance of MenB-4C antigen variants in invasive meningococcal disease isolates (*79*).

## Serogroups A, C, W, and Y Meningococcal Vaccines

### MenACWY-D (Menactra)

MenACWY-D was first licensed in the United States in 2005. Clinical trials have demonstrated immunogenicity of MenACWY-D among persons aged 9 months–55 years, although antibody waning is observed during the 3–5 years after primary vaccination (*67*,*80*–*104*). Booster vaccination elicits a robust immune response, and data in adolescents demonstrate persistence for at least 4 years after a booster dose (*105*,*106*). Clinical trials have demonstrated an acceptable safety profile, with injection site pain and erythema as the most common local reactions; irritability and drowsiness are the most common systemic adverse events among infants and children, and myalgia, headache, and fatigue are the most common systemic adverse events among adolescents and adults (*67*,*80*,*81*,*85*,*87*–*90*,*92*–*96*,*98*–*103*). Most adverse events are mild to moderate and resolve within 3 days. Early postlicensure surveillance raised the concern of a potential risk for Guillain-Barré syndrome (GBS), but subsequent evaluations have not identified an increased risk for GBS after MenACWY-D vaccination (*107*–*110*). No other vaccine safety concerns have been identified in postlicensure surveillance (*111*–*114*).

#### MenACWY-D Immunogenicity

##### Infants and Children

In clinical trials among infants who received MenACWY-D as a 2-dose series at ages 9 and 12 months, 89%–96% achieved an hSBA titer ≥1:8 against serogroup A, ≥98% against serogroup C, 81%–92% against serogroup W, and 95%–97% against serogroup Y 1 month after completion of the series (*80*,*98*). Administration of MenACWY-D simultaneously with routine vaccines did not result in reduced immune responses to meningococcal serogroups A, C, W, or Y or measles, mumps, rubella, or varicella antigens; however, when MenACWY-D was administered simultaneously with seven-valent pneumococcal conjugate vaccine (PCV7) (Prevnar), noninferiority criteria were not met for three of seven pneumococcal serotypes (*98*). By 3 years after primary series completion, substantial MenACWY-D waning occurred, with 13%–46% of recipients having an hSBA titer ≥1:8 across serogroups, although this proportion increased to ≥98% 1 month after a single booster dose (*80*).

Among toddlers receiving a 2-dose primary series at ages 12 and 15 months, 85% achieved an hSBA titer ≥1:8 against serogroup A and ≥96% against serogroups C, W, and Y at 1 month after the primary series (*80*). In another study in which MenACWY-D was administered at ages 12 and 18 months, ≥96% achieved rSBA titers ≥1:8 for all serogroups (*95*). In this study, administration of MenACWY-D simultaneously with routine vaccines did not result in reduced immune responses to meningococcal serogroups A, C, W, or Y or tetanus, diphtheria, pertussis, poliovirus, or *H. influenzae* type b antigens.

Among children aged 2–10 years, the rate of seroresponse (defined as a greater than fourfold rise in hSBA or a titer ≥1:8 among persons with baseline titers <4) was highest for serogroup A (80%) and lower for serogroups C, W, and Y (42%–57%) at 1 month after a single dose (*88*). Studies using rSBA demonstrated a higher proportion of seroresponse across serogroups (≥86%) using different thresholds (either a greater than fourfold rise in titers among persons with baseline titers <1:8 or a titer ≥1:8) (*91*,*93*,*96*,*104*). Among children aged 4–6 years, administration of MenACWY-D simultaneously with routine vaccines did not result in reduced immune response to meningococcal serogroups A, C, or W or diphtheria, tetanus, or poliovirus antigens; however, the noninferiority criteria were not met for serogroup Y and one pertussis antigen (anti-fimbriae) (*67*). Because no clinical correlates of protection are available for pertussis antigens, the clinical significance of this finding is unknown. When MenACWY-D was administered 30 days after diphtheria and tetanus toxoids and acellular pertussis (DTaP) vaccine (DAPTACEL), significantly lower geometric mean titers (GMTs) were observed for all meningococcal serogroups (*67*). Among children aged 4–6 years vaccinated previously at age 2–3 years, the proportions maintaining an rSBA titer ≥1:128 were 75%, 52%, 61%, and 90% for serogroups A, C, W, and Y, respectively (*97*).

##### Adolescents and Adults

Among adolescents and adults aged 10–55 years, 64%–71% achieved an hSBA titer ≥1:8 against serogroup A, 72%–99% against serogroup C, 64%–90% against serogroup W, and 39%–82% against serogroup Y at 1 month after vaccination with a single dose (*81*,*87*,*89*,*99*). In studies assessing immunogenicity using rSBA, ≥80% and ≥88% achieved seroprotection across serogroups when the thresholds of ≥1:128 and ≥1:8 were used, respectively (*85*,*90*,*92*,*102*,*104*). Administration of MenACWY-D simultaneously with routine vaccines did not result in reduced immune responses to meningococcal serogroups A, C, W, or Y or tetanus, diphtheria, pertussis, human papillomavirus (HPV), MenB-FHbp, or typhoid antigens (*67*,*94*,*100*–*103*).

Persistence studies conducted among adolescents and adults demonstrated antibody waning after primary vaccination; however, serogroup-specific degree of waning varied between the studies. In one study, antibody waning was observed for all serogroups, particularly serogroup A, by 22 months postvaccination and titers remained stable thereafter at 3 and 5 years postvaccination; 21%–34% of recipients achieved an hSBA titer ≥1:8 for serogroup A, 58%–62% for serogroup C, 71%–74% for serogroup W, and 53%–54% for serogroup Y between 22 months and 5 years postvaccination (*83*,*84*,*86*). In another study, antibody waning was observed by 4–6 years postvaccination but was more marked for serogroups C and Y (44% and 39% achieved an hSBA titer ≥1:8, respectively) compared with serogroups A and W (65% and 69%, respectively) (*105*). In a separate study, serogroup A waning was most pronounced, although for the other serogroups the proportion of recipients with hSBA titers ≥1:8 was higher than that observed in previously mentioned studies and was stable at 1, 3, and 5 years postvaccination with 32%–44% seroprotected against serogroup A, 73%–81% against serogroup C, 76%–85% against serogroup W, and 87%–91% against serogroup Y (*82*). Thus, although antibody waning after primary vaccination of adolescents and adults was observed across studies, time points assessed and patterns of waning by serogroup were not consistent. In a study of adolescents who received a booster dose of MenACWY-D, ≥99% achieved hSBA titers ≥1:8 against all serogroups at 1 month postvaccination; this proportion remained ≥90% 4 years later (*105*,*106*).

#### MenACWY-D Safety

##### Clinical Trials

Among infants vaccinated at ages 9 and 12 months and toddlers vaccinated at ages 12 months and 15 or 18 months, the most commonly reported local reactions after either of the doses were injection site pain (35%–59%) and erythema (23%–43%) (*80*,*95*,*98*). The most commonly reported systemic adverse events were irritability (49%–72%) and drowsiness (27%–44%); fever was reported in 11%–50% of recipients. Adverse events among infants were similar when MenACWY-D was administered alone or simultaneously with other vaccines (*98*). After receipt of a booster dose 3 years after vaccination as an infant, rates of local and systemic adverse events were similar to those observed for the primary series (*115*). Similar adverse events were observed among children aged 2–10 years after a single dose, although typically at a slightly lower rate; injection site pain (32%–48%), induration (11%–22%), and erythema (10%–30%) were the most commonly reported local reactions, and drowsiness (9%–26%), irritability (7%–35%), and fever (2%–11%) were the most common systemic adverse events (*67*,*88*,*93*,*96*).

Among adolescents and adults aged 11–55 years who received a single dose, injection site pain (31%–69%) was the most common local reaction, followed by induration (9%–20%), erythema (3%–20%), and swelling (1%–14%) (*81*,*85*,*87*,*89*,*90*,*92*,*99*,*102*). Myalgia (15%–26%), headache (11%–45%), fatigue or malaise (10%–28%), and diarrhea or other gastrointestinal symptoms (11%–17%) were the most commonly reported systemic adverse events; fever was observed in <8%. Similar types and rates of adverse events were observed after a booster dose administered 4 years later (*105*). In general, MenACWY-D administered simultaneously with HPV vaccine, tetanus and reduced diphtheria toxoids and acellular pertussis vaccine (Tdap), MenB-FHbp, or typhoid vaccines was well tolerated, although rates of some adverse events (e.g., headache and fatigue) were slightly higher with simultaneous administration compared with MenACWY-D administered alone (*67*,*94*,*100*–*103*). Across age groups, whether MenACWY-D was administered alone or simultaneously with other vaccines, adverse events were mild to moderate and typically resolved within 3 days.

##### Postlicensure Safety Monitoring

After licensure of the vaccine in 2005, several cases of GBS after MenACWY-D vaccination were reported to VAERS (*116*). ACIP reviewed the available data and determined that the benefits of meningococcal vaccination outweighed the small potential increased risk for GBS (*107*). By 2010, two retrospective evaluations had been conducted in which no GBS cases were observed in the 6 weeks after 2.3 million doses of MenACWY-D were administered (*108*,*110*). The excess risk for GBS after vaccination, if it exists, is estimated to be <0.66 cases per 1 million adolescents vaccinated (*110*). In 2010, ACIP voted to remove the precaution for persons with a history of GBS from the ACIP recommendations, although it continues to be listed as a precaution in the package insert (*16*,*67*). Evaluations of VSD data through 2014 and VAERS data through 2016 have since been conducted, identifying no new GBS-related concerns (CDC, unpublished data, 2020).

In addition to assessing risk for GBS, data from VAERS and VSD have been evaluated to assess for other potential postvaccination adverse events. In a comprehensive review of VAERS reports received from 2005 through June 2016, during which approximately 70 million MenACWY-D doses were distributed, no new safety concerns were identified (CDC, unpublished data, 2020). A total of 13,075 adverse events related to MenACWY-D were reported, of which 846 (6.5%) were serious. Reports predominantly related to adolescents aged 11–18 years simultaneously vaccinated with MenACWY-D and other vaccines. The most commonly reported events were injection site erythema, fever, and headache, consistent with findings from clinical trials.

An analysis of VSD during 2005–2014, when 1.4 million doses, including 245,000 booster doses, were administered, was conducted to evaluate prespecified outcomes (CDC, unpublished data, 2020). Although rates of syncope and medically attended fever increased after MenACWY-D vaccination, no new safety concerns were identified. Furthermore, tree-temporal scan data mining through VSD of primary doses administered to 1.2 million adolescents aged 11–18 years during the same period identified no new or unexpected adverse events within 42 days after MenACWY-D administration (*114*). Several smaller studies, including VSD- and manufacturer-sponsored studies conducted during 2005–2014, similarly did not identify any additional safety concerns for MenACWY-D among infants, children, adolescents, or adults (*111*–*113*).

### MenACWY-CRM (Menveo)

MenACWY-CRM was first licensed in the United States in 2010. MenACWY-CRM has been demonstrated to be immunogenic among persons aged 2 months–55 years (*88*,*89*,*99*,*117*–*130*). Antibody waning occurs by 3–5 years after primary vaccination, and booster vaccination elicits a robust immune response (*83*,*84*,*131*–*133*). No consistent or clinically relevant concerns about MenACWY-CRM administered simultaneously with other vaccines have been identified (*134*–*136*). Clinical trials have demonstrated an acceptable safety profile, with injection site pain and erythema as the most common local reactions (*88*,*118*–*124*,*126*,*127*,*129*,*137*). Irritability and sleepiness were the most commonly reported systemic adverse events among infants and toddlers. Among children, irritability, myalgia, headache, and sleepiness were the most commonly reported systemic adverse events, whereas myalgia, headache, and fatigue were the most commonly reported systemic adverse events among adolescents and adults. Most adverse events were mild to moderate and resolved within 3 days. One study observed an increased risk for Bell’s palsy among adolescents after MenACWY-CRM vaccination, although this was based on a small number of cases and the importance of this finding is uncertain (*138*). No additional safety concerns have been identified in postlicensure surveillance, although administration errors appear more common than with other vaccines, predominantly because of the need to reconstitute the vaccine using lyophilized and liquid components (*139*,*140*).

#### MenACWY-CRM Immunogenicity

##### Infants and Children

Among infants vaccinated at ages 2, 4, and 6 months with MenACWY-CRM and routine vaccines, 76%–89% achieved an hSBA titer ≥1:8 for serogroup A and ≥94% for serogroups C, W, and Y 1 month after the third dose (*119*,*124*,*128*,*130*,*141*). Antibody titers waned by age 12 months, particularly for serogroup A. Following the fourth dose in the infant series at age 12–17 months, the proportions of infants with an hSBA titer ≥1:8 were 89%–96% for serogroup A and ≥95% for serogroups C, W, and Y. Administration of MenACWY-CRM simultaneously with routine vaccines did not result in reduced immune responses to meningococcal serogroups A, C, W, or Y or diphtheria, tetanus, hepatitis B, poliovirus, measles, mumps, rubella, varicella, *H. influenzae* type b, or most pneumococcal antigens (*136*). For the few pneumococcal serotypes for which noninferiority criteria were not met, results were not consistent across studies and age of administration (*136*). Noninferiority was not consistently met for pertussis antigens across studies when MenACWY-CRM was administered with routine vaccines; however, the clinical relevance is unclear because of the lack of clinical correlates of protection for pertussis antigens. By age 2 years, or 1 year after completion of a 4-dose MenACWY-CRM series, 88%–89% achieved an hSBA titer ≥1:8 for serogroups W and Y, 61% for serogroup C, and 39% for serogroup A (*141*). By age 40 months, most children still had an hSBA titer ≥1:8 for serogroups W and Y (76% and 67%, respectively), although this proportion was only 34% for serogroup C and 10% for serogroup A. By age 60 months, similar but slightly lower proportions were observed; following a single booster dose, ≥96% of recipients achieved hSBA titers ≥1:8 across all serogroups.

After a 2-dose series among older infants and toddlers at either ages 7–9 months and 12 months or 12 and 15 months, the proportions who achieved an hSBA titer ≥1:8 1 month after the second dose were 88%–97% for serogroup A and ≥96% for serogroups C, W, and Y (*125*,*130*). Administration of MenACWY-CRM simultaneously with routine vaccines did not result in reduced immune responses to meningococcal serogroups A, C, W, or Y or measles, mumps, rubella, or varicella antigens (*136*). Among children who received the 2-dose primary series at ages 12–13 and 15 months, serogroup-specific antibody waning patterns similar to those among infants also were observed; however, the proportion of recipients with an hSBA titer ≥1:8 was higher at age 40 months (85%, 79%, 55%, and 31% for serogroups W, Y, C, and A, respectively) among this group (*131*). Similar results were observed by age 60 months; after a single booster, all subjects achieved hSBA titers ≥1:8 for all serogroups (*131*).

Among children who received a single dose at age 2–10 years, the proportions who achieved hSBA titers ≥1:8 (≥1:4 in one study) were 72%–89% for serogroup A, 68%–94% for serogroup C, 90%–96% for serogroup W, and 65%–91% for serogroup Y (*88*,*118*,*120*,*121*,*123*,*126*). No data are available on MenACWY-CRM administered simultaneously with routine vaccines among this age group. Twelve months after the primary dose, the proportion with seroprotective titers declined, particularly for serogroups A and C (*118*,*123*). Five years after a primary dose administered at age 2–10 years, 14%–22% of recipients remained seroprotected against serogroup A, 32%–56% against serogroup C, 74%–80% against serogroup W, and 48%–53% against serogroup Y; these proportions were lower among those vaccinated at age 2–5 years compared with age 6–10 years. One month after a single booster dose, all recipients achieved hSBA titers ≥1:8 for all serogroups (*132*).

##### Adolescents and Adults

One month after a single MenACWY-CRM dose among adolescents and adults aged 11–75 years, 66%–92% of recipients achieved an hSBA titer ≥1:8 for serogroup A, 79%–98% for serogroup C, 84%–99% for serogroup W, and 79%–96% for serogroup Y (*89*,*99*,*117*,*120*–*122*,*126*,*127*,*129*). In one study that reported immunogenicity separately for adults aged ≥55 years, those aged 56–65 years had results similar to those aged 19–55 years (*129*). Administration of MenACWY-CRM simultaneously with other vaccines did not result in reduced immune responses to meningococcal serogroups A, C, W, or Y or tetanus, diphtheria, HPV, hepatitis A, hepatitis B, typhoid, yellow fever, Japanese encephalitis, or rabies antigens (*117*,*135*,*142*–*146*). After simultaneous administration of MenACWY-CRM and MenB-4C, a robust immune response to meningococcal serogroups A, C, W, and Y and to select meningococcal serogroup B strains was observed, although the majority of persons had high prevaccine hSBA titers across serogroups (*134*). Noninferiority criteria were not met for two pertussis antigens (i.e., pertussis toxoid and pertactin) when MenACWY-CRM and Tdap were administered simultaneously, although the clinical relevance of this is unclear (*135*).

By 12–22 months postvaccination, substantial antibody waning was observed for serogroup A, though the majority of recipients remained seroprotected for serogroups C, W, and Y (*86*,*122*). After this initial decline, hSBA titers remained relatively stable at 3 and 5 years postvaccination, with 28%–32% of recipients having an hSBA titer ≥1:8 against serogroup A, 59%–76% against serogroup C, 72%–82% against serogroup W, and 64%–76% against serogroup Y (*83*,*133*). One month after a single MenACWY-CRM booster dose administered at 3–6 years after the primary dose, ≥94% of subjects achieved an hSBA titer ≥1:8 across all serogroups. Booster vaccination elicited a robust immune response whether MenACWY-CRM or MenACWY-D was used for the primary dose (*83*,*147*). By 2 years after the booster dose, the proportion of recipients with an hSBA titer ≥1:8 decreased to 79% for serogroup A but remained at ≥95% for serogroups C, W, and Y (*84*).

#### MenACWY-CRM Safety

##### Clinical Trials

Among infants and toddlers vaccinated with a MenACWY-CRM series (4-dose and 2-dose series, respectively) administered with routine vaccines, injection site pain (19%–39%) and erythema (12%–22%) were the most common local reactions after the third or fourth infant doses and second toddler dose (*119*,*124*,*137*,*141*). Irritability (36%–50%), sleepiness (22%–31%), and decreased appetite (15%–20%) were the most common systemic adverse events; fever was reported in 5%–9% of recipients. Reactogenicity among infants and toddlers vaccinated with a 4- or 2-dose series, respectively, did not increase with subsequent MenACWY-CRM doses. Among children aged 2–10 years vaccinated with a single dose, injection site pain (<40%) and erythema (<32%) were the most commonly reported local reactions (*88*,*118*,*120*,*121*,*123*,*126*). Irritability was reported in 7%–26%, myalgia in <29%, headache in <21%, and fatigue in <21%. Adverse events were similar whether vaccination was administered at ages 2–5 or 6–10 years. Adverse events were similar after a booster dose administered 5 years after primary vaccination (*132*).

Among adolescents and adults aged 11–75 years who received a single MenACWY-CRM dose, injection site pain occurred in 8%–54%, erythema in <39%, and induration in <24%. Commonly reported systemic adverse events include headache (8%–41%), myalgia (<43%), and fatigue (3%–23%) (*99*,*120*–*122*,*126*,*127*,*129*,*148*). When MenACWY-CRM was administered simultaneously with HPV and Tdap vaccines, headache, malaise, myalgia, and arthralgia occurred more often than when MenACWY-CRM was administered alone (*117*). In addition, adverse events after a booster dose administered 4–6 years after primary vaccination were similar to those among persons receiving a first dose. Across age groups, whether MenACWY-CRM was administered alone or simultaneously with other vaccines, adverse events were mild to moderate and typically resolved within 3 days.

##### Postlicensure Safety Monitoring

In a manufacturer-sponsored cohort study of approximately 49,000 vaccinated adolescents aged 11–21 years with a self-controlled case series analysis, a statistically significant increased risk for Bell’s palsy during the 84 days after vaccination was observed when MenACWY-CRM was administered simultaneously with other vaccines but not when MenACWY-CRM was administered alone (*138*). However, this finding was based on only eight patients, most of whom received simultaneous vaccine administration, and several were noted to have had conditions or infections that might precede Bell’s palsy. Thus, the importance of this finding remains uncertain. No other safety signals were observed among the other predefined events of interest in this evaluation (*138*). No increased risk for Bell’s palsy or any other new safety concerns were observed in smaller studies conducted in the same health system among children aged 2 months–10 years (*140*,*149*).

A comprehensive review of VAERS reports from 2010 through 2015, during which 8.2 million MenACWY-CRM doses were distributed, was conducted with no new safety concerns identified (*139*). A total of 2,614 reports about MenACWY-CRM were received, primarily related to adolescents aged 11–18 years in whom MenACWY-CRM was administered simultaneously with other vaccines. Reported adverse events were consistent with the findings from prelicensure studies. The reporting rate of GBS or Bell’s palsy was proportionate to the rate reported for other vaccines. However, administration errors were reported more commonly for MenACWY-CRM, predominantly because of administration of only one component (most commonly the liquid component) rather than reconstituting the vaccine by mixing the liquid and lyophilized components together before administration.

### MenACWY-TT (MenQuadfi)

MenACWY-TT was first licensed in the United States in 2020 for the prevention of meningococcal disease caused by serogroups A, C, W, and Y in persons aged ≥2 years (*68*). As a result, relatively limited data on MenACWY-TT safety and immunogenicity are available compared with other licensed meningococcal conjugate vaccines. MenACWY-TT has been administered to nearly 5,000 persons aged ≥2 years to date through clinical trials, with demonstrated immunogenicity in this age group and elicitation of a boost response among adolescents vaccinated with MenACWY-TT who previously received MenACWY-D or MenACWY-CRM (*68*,*150*–*155*). No clinically relevant concerns about MenACWY-TT administered simultaneously with HPV or Tdap vaccines among adolescents have been identified (*151*). Clinical trials have demonstrated an acceptable safety profile, with injection site pain as the most common local adverse event, and myalgia, headache, and malaise as the most commonly reported systemic adverse events across age groups (*68*,*150*–*155*). Most adverse events were mild to moderate (*68*,*150*–*155*).

#### MenACWY-TT Immunogenicity

##### Infants and Children

Because MenACWY-TT is currently only licensed for persons aged ≥2 years in the United States, immunogenicity and safety data for children aged <2 years are not presented in this report. Among children who received a single dose at age 2–9 years, the proportions who achieved hSBA titers ≥1:8 1 month after vaccination were 86% for serogroup A, 98% for serogroup C, 95% for serogroup W, and 99% for serogroup Y (*155*). MenACWY-TT seroresponse rates were demonstrated to be noninferior to those observed for MenACWY-CRM (*155*). No data are available on MenACWY-TT administered simultaneously with routine vaccines or on persistence of the immune response to MenACWY-TT among this age group; data will be reviewed as they become available to inform vaccine recommendations.

##### Adolescents and Adults

One month after a single MenACWY-TT dose among adolescents and adults aged 10–55 years, the proportions who achieved hSBA titers ≥1:8 were 94%–96% for serogroup A, 94%–99% for serogroup C, 95%–99% for serogroup W, and 97%–99% for serogroup Y (*151*,*154*). Among adults aged ≥56 years, these proportions were 89%–94%, 75%–90%, 77%–80%, and 81%–92% for serogroups A, C, W, and Y, respectively (*152*,*153*). Across these age groups, MenACWY-TT seroresponse rates were noninferior to those of the comparator meningococcal vaccines (*68*,*151*,*153*). MenACWY-TT administered simultaneously with HPV and Tdap vaccines in adolescents did not result in reduced immune responses to meningococcal serogroups or tetanus, diphtheria, or HPV antigens (*151*). Noninferiority criteria were not met for three pertussis antigens when MenACWY-TT and Tdap were administered simultaneously, although the clinical relevance of this is unclear. No data are available on persistence of the immune response to MenACWY-TT. When available, data will be reviewed to inform booster dose recommendations for persons primed with MenACWY-TT. Among adolescents and adults aged ≥15 years primed with MenACWY-D or MenACWY-CRM 4–10 years previously, >99% achieved an hSBA titer ≥1:8 across all serogroups at 1 month after booster vaccination with MenACWY-TT (*150*).

#### MenACWY-TT Safety

##### Clinical Trials

Among children aged 2–9 years vaccinated with a single MenACWY-TT dose, injection site pain occurred in 39%, erythema in 23%, and swelling in 14%; systemic adverse events included malaise in 21%, myalgia in 20%, headache in 13%, and fever in 2% within 7 days of vaccine administration (*68*). Among adolescents and adults aged 10–55 years, the most common local adverse event was injection site pain (35%–45%); erythema and swelling occurred in 5% and 4%–5%, respectively (*68*,*151*). Systemic adverse events included myalgia (27%–36%), headache (27%–30%), and malaise (19%–26%); fever occurred in 1% (*68*,*151*). Rates of local and systemic adverse events were typically similar in adults aged ≥56 years compared with other age groups: injection site pain (26%–31%), erythema (5%–12%), swelling (5%–8%), myalgia (22%–35%), headache (19%–24%), malaise (15%–22%), and fever (2%). When MenACWY-TT was administered simultaneously with HPV and Tdap vaccines in adolescents, rates of local and systemic adverse events were typically similar to those when MenACWY-TT was administered alone, although myalgia occurred more frequently (*151*). In addition, adverse events after a MenACWY-TT booster dose administered 4–10 years after primary vaccination with either MenACWY-D or MenACWY-CRM were similar to those among persons receiving a first MenACWY-TT dose (*150*). Across age groups, whether MenACWY-TT was administered alone or simultaneously with other vaccines, adverse events were mild to moderate (*68*,*150*–*155*).

##### Postlicensure Safety Monitoring

Given the recent licensure of MenACWY-TT, no postlicensure data were available at the time of publication of this report. Data from VAERS and VSD will be monitored as part of postlicensure safety monitoring. (See Reporting of Vaccine Adverse Events for information on how to report MenACWY-TT adverse events to VAERS.)

### MenACWY Vaccine Immunogenicity and Safety in Persons with Underlying Medical Conditions

Complement-mediated bactericidal activity is important for protection against meningococcal disease; opsonophagocytic killing elicited by meningococcal antibodies is another defense against infection and is the presumed primary mechanism for vaccine-induced protection against meningococcal disease among persons with complement deficiency (*35*,*156*). No data about immunogenicity of U.S.-licensed MenACWY vaccines (MenACWY-D, MenACWY-CRM, or MenACWY-TT) are available for persons with complement deficiency. Antibody titers after vaccination with MPSV4, a vaccine that is no longer available in the United States, are similar among persons with late complement deficiency compared with healthy persons and the antibodies produced are capable of eliciting opsonophagocytosis; however, antibody titers might wane more rapidly among persons with complement deficiency and higher antibody levels might be needed for opsonophagocytosis to function (*35*,*157*–*160*). Data are lacking to establish the efficacy of meningococcal conjugate vaccines among persons with complement deficiency. Thus, persons with complement deficiency are at increased risk for meningococcal disease even if they develop antibodies postvaccination (*66*–*68*).

Limited data are available about efficacy of meningococcal vaccines among persons taking complement inhibitors. However, some studies suggest that opsonophagocytic killing of meningococci in the presence of eculizumab in sera from persons vaccinated with MenACWY either does not occur or is insufficient to prevent meningococcal proliferation (*161*). In addition, reports of meningococcal disease despite recent vaccination among persons taking eculizumab indicate that meningococcal vaccines do not provide complete protection among persons taking complement inhibitors, even if antibodies develop after vaccination (*42*,*66*–*68*,*161*).

Although no data are available for U.S.-licensed MenACWY vaccines (MenACWY-D, MenACWY-CRM, or MenACWY-TT), adults with anatomic asplenia had a reduced immunologic response compared with healthy persons after 1 dose of a serogroup C meningococcal conjugate vaccine; after a second dose, most persons achieved seroprotection (*162*–*164*). Among children and adolescents vaccinated with a conjugate MenACWY-TT vaccine licensed outside the United States, similar immune responses were observed in children with functional or anatomic asplenia compared with healthy controls after each of 2 doses (*165*). However, antibodies appear to wane rapidly after serogroup C meningococcal conjugate vaccination among children with functional asplenia due to sickle cell disease, particularly among those who received primary vaccination at age <2 years (*166*).

Among adolescents with HIV infection, immunogenicity to MenACWY-D is reduced compared with adolescents without HIV infection. By 4 weeks postvaccination with a single dose, 52%–73% of HIV-infected adolescents had a greater than fourfold increase in rSBA across the meningococcal serogroups. Lower CD4 percentage, higher viral load, and a more advanced clinical stage were inversely associated with seroprotection against serogroup C (*167*). By 72 weeks subsequent to a second dose, a significantly greater proportion of adolescents with a CD4 percentage ≥15% had seroprotective rSBA titers, although this proportion was lesser for serogroup C than other serogroups, compared with those with a CD4 percentage of <15%, among whom seroprotection rates for all serogroups was reduced (*168*). Among children aged 2–10 years with HIV infection and a CD4 percentage ≥25%, antibody titers against serogroup C waned substantially by 72 weeks after vaccination (*169*). Similar trends were observed subsequent to vaccination of HIV-infected children and adolescents with serogroup C meningococcal conjugate vaccine (*170*–*173*).

Although data are limited, vaccination of persons with asplenia or HIV infection appears to be safe and well tolerated, with similar types of adverse events as reported among healthy controls or during clinical trials (*165*,*167*,*169*,*171*). Among HIV-infected children and adolescents vaccinated with MenACWY-D, rates of adverse events typically were lower than those reported during clinical trials of healthy children and adolescents, although these lower adverse event rates were not consistently observed among those vaccinated with a serogroup C meningococcal conjugate vaccine (*80*,*167*,*169*–*171*). Among children with asplenia who received a conjugate MenACWY-TT vaccine licensed outside the United States, an acceptable safety profile was observed among all age groups, although higher rates of adverse events were reported compared with healthy controls; however, the small study size limits the interpretation of this finding (*165*,*170*,*171*).

### MenACWY Vaccines in Pregnant Women

Adverse outcomes (e.g., spontaneous abortion or birth defects) are risks for all pregnancies, occurring in approximately 15%–20% and 3%, respectively, of clinically recognized pregnancies in the United States (*174*,*175*). Although evidence is limited, rates of these outcomes after MenACWY vaccination during pregnancy are consistent with the estimated background rates, and no additional concerning maternal or neonatal safety patterns have been identified (*66*,*67*,*112*,*139*,*176*,*177*).

No controlled trials have been conducted to specifically assess the safety of meningococcal vaccination among pregnant women and birth outcomes of vaccinated women. However, among approximately 2,000 pregnant Malian women vaccinated during the third trimester with MenACWY-D as a control group in an influenza vaccine trial, rates of local and systemic adverse events were lower than those observed during MenACWY-D clinical trials of adolescents and adults and serious obstetric and nonobstetric adverse events were rare, with similar rates between MenACWY-D and influenza vaccination groups (*176*). In the MenACWY-D vaccinated group, 98% of pregnancies resulted in live births, and among infants, 0.3% had low birthweight and 0.2% had a congenital malformation; no differences in these outcomes were observed among women who received influenza vaccine.

Among approximately 5,000 adolescent or adult females enrolled in MenACWY clinical trials, pregnancy was reported in 43 women during the 6 months postvaccination (37 who received MenACWY-CRM and six who received MenACWY-D) (*66*,*67*). Of these, seven (19%) MenACWY-CRM recipients reported spontaneous abortion (estimated dates of conception were 5 days before vaccination for one woman, 6–17 weeks postvaccination for five women, and 6 months postvaccination for one woman). Congenital anomaly (hydrocephalus) was reported in the infant of one MenACWY-D recipient with an estimated conception date 15 weeks after vaccination.

Although data are limited, no concerning safety signals have been identified through postlicensure surveillance. In reviews of VAERS, 127 pregnancy-associated reports were identified during the periods evaluated: 113 for MenACWY-D (2005–2011) and 14 for MenACWY-CRM (2010–2015); the differences in number of reports by vaccine type probably reflect differences in numbers of doses administered during these periods (*139*,*177*). The majority of vaccine administrations occurred during the first trimester. Among the 113 pregnant women who received MenACWY-D, spontaneous abortion was reported in 17% and congenital anomaly was reported in <1% of VAERS reports (*177*). Following MenACWY-CRM vaccination in pregnancy, only three VAERS reports had information on birth outcome, with no adverse events reported (*139*). Among patients in a large health care organization, one spontaneous abortion was identified among 18 MenACWY-D exposures during pregnancy with known outcome (*112*).

Manufacturers of MenACWY vaccines maintain registries that monitor pregnancy outcomes of women exposed to MenACWY during pregnancy. Among 87 pregnant women exposed to MenACWY-D during 2005–2016 from 30 days before or at any time during pregnancy who had known pregnancy outcome and who were enrolled in the registry before outcome being known, spontaneous abortion was reported in 7% and major congenital anomalies in 2% (*67*). Among 82 pregnant women exposed to MenACWY-CRM during 2014–2017 from 28 days before or at any time during pregnancy who had known outcome, spontaneous abortion was reported in 12% and congenital anomaly in 4% (GlaxoSmithKline, unpublished data, 2019). No information is available from the MenACWY-TT pregnancy registry because of the recent licensure of the vaccine.

### Effectiveness of MenACWY Vaccines

Overall vaccine effectiveness of a single dose of MenACWY-D against meningococcal disease caused by serogroups A, C, W, or Y among adolescents in the United States is estimated at 69% (95% confidence interval [CI]: 51%–80%) in the 8 years after vaccination: 77% (95% CI: 57%–88%) against serogroup C and 51% (95% CI: 1%–76%) against serogroup Y (*178*). Effectiveness was 79% (95% CI: 49%–91%) in the first year but decreased to 69% (95% CI: 44%–83%) 1 to <3 years postvaccination and 61% (95% CI: 25%–79%) 3 to <8 years postvaccination. No data are available on the effectiveness of MenACWY-CRM or MenACWY-TT.

#### Vaccination and Meningococcal Disease Incidence

Measuring the association between adolescent MenACWY vaccination on rates of meningococcal disease has been challenging because of the low and decreasing incidence of meningococcal disease among all age groups. However, from MenACWY introduction through 2017, adolescents experienced the greatest percentage decreases (>90%) in meningococcal disease incidence due to serogroups C, W, or Y combined compared with other age groups (*179*). In the setting of 85% coverage with at least 1 dose of MenACWY-D or MenACWY-CRM among U.S. adolescents aged 13–17 years and 44% coverage with at least 2 doses among adolescents aged 17 years by 2017, a twofold to threefold increase in the rate of decline in incidence was observed during the postvaccination period compared with the prevaccination period among adolescents, suggesting that vaccination with MenACWY-D or MenACWY-CRM is associated with reductions in disease rates in adolescents (*179*,*180*). No data are available for MenACWY-TT.

#### Vaccination and Oropharyngeal Carriage

Although vaccination with a serogroup C meningococcal conjugate vaccine in Europe and a serogroup A meningococcal conjugate vaccine in sub-Saharan Africa has been associated with reductions in oropharyngeal carriage of these *N. meningitidis* serogroups and resulted in herd immunity in the population (*181*–*183*), data are limited for MenACWY vaccines. In the United States, carriage prevalence of meningococcal serogroups C, W, or Y combined among college students in the setting of high MenACWY vaccination coverage is now extremely low (<1%); however, no direct evidence exists that this low prevalence is a result of vaccination (*184*–*186*). In a small observational study of Polish military recruits, those vaccinated with a MenACWY vaccine 1–3 years earlier had lower rates of meningococcal carriage compared with unvaccinated recruits (*187*). In a randomized controlled trial of United Kingdom university students, those who received MenACWY-CRM had significantly lower carriage prevalence than controls for serogroup Y (39% carriage reduction) and serogroups C, W, and Y combined (36% carriage reduction) at 2 months postvaccination, although no differences in carriage acquisition rates were observed (*188*). In contrast, in a study conducted in a different United Kingdom university population vaccinated with MenACWY-CRM in response to rapid expansion of a serogroup W clone in England, serogroup W carriage of this clone increased despite relatively high vaccination coverage (*189*). However, because carriage acquisition among university students is known to rapidly increase at the beginning of the academic year (*190*), the majority of serogroup W transmissions might have occurred simultaneously with vaccination activities (i.e., during September).

### Cost-Effectiveness of MenACWY Vaccines

Cost-effectiveness of MenACWY vaccines in the United States was last assessed in 2010 using Monte Carlo simulation (*191*). In this evaluation, cost per quality-adjusted life year (QALY) of vaccinating at ages 11 and 16 years was similar to vaccinating at either age 11 or 15 years ($212,000–$256,000), although the estimated number of cases and deaths averted among the vaccinated cohort was substantially higher with a 2-dose strategy (184 and 22, respectively) compared with a single-dose strategy (94–115 and 11–14, respectively) (*16*).

## Serogroup B Meningococcal Vaccines

### MenB-FHbp (Trumenba)

MenB-FHbp is only licensed for persons aged 10–25 years in the United States; therefore, immunogenicity and safety data for children aged <10 years are not presented in this report. Available data for this age group have recently been summarized elsewhere (*192*).

Clinical trials have demonstrated that although vaccination of adolescents and young adults with either a 2- or 3-dose schedule of MenB-FHbp is immunogenic, antibody titers wane substantially by 1 year postvaccination and then remain stable for up to 4 years (*94*,*193*–*199*). Subsequent to booster vaccination 4 years after primary series completion, a robust response is observed and persistence at 26 months after the booster dose is superior to the response at a comparable period after primary series completion for both 2-dose and 3-dose primary series recipients (*19*,*200*). No clinically relevant concerns related to MenB-FHbp administered simultaneously with other vaccines have been identified (*94*,*196*,*201*). MenB-FHbp is safe and well tolerated, although more reactogenic than MenACWY (*94*,*194*–*199*,*201*). In clinical trials, the most common local reactions were injection site pain, induration, and erythema, and the most common systemic adverse events were headache, fatigue, and myalgia. Symptoms typically resolved within 5 days (*94*,*194*–*199*,*201*). Adverse events reported through postlicensure safety surveillance are consistent with the clinical trial data, and no new safety concerns have been identified (*202*).

#### MenB-FHbp Immunogenicity

Clinical trials for MenB-FHbp immunogenicity against four reference strains, each expressing an FHbp antigen different from those included in the vaccine, among persons aged 10–25 years were conducted using several dosing schedules, including the licensed formulations of a 3-dose schedule (0, 1, and 6 months or 0, 2, and 6 months) and a 2-dose schedule (0 and 6 months). By 1 month after completion of either of the 3-dose schedules, the proportion of persons with hSBA titers ≥1:8 or at or above the lower limit of quantification (LLOQ) of the assay (LLOQ: ≥1:8 or ≥1:16 depending on the strain) was 91%–98% for test strain A22, ≥99% for A56, 81%–95% for B24, 86%–96% for B44, and 84%–94% for the composite response (i.e., response against all test strains) (*94*,*193*–*199*). The proportions of persons with seroprotective hSBA titers were similar between 0-, 1-, and 6-months and 0-, 2-, and 6-months schedules. In the study that assessed the 2-dose schedule (0 and 6 months), the proportion with hSBA titers ≥LLOQ was 97% for A22, 99% for A56, 81% for B24, 78% for B44, and 77% for the composite response (*182*,*198*). Administration of MenB-FHbp simultaneously with other vaccines did not result in reduced immune response to MenB-FHbp antigens, meningococcal serogroups A, C, W, or Y or diphtheria, tetanus, or pertussis antigens (*94*,*201*). Noninferiority criteria were not met for HPV 18 antigen, although the GMTs were high and for each of the four HPV types in the quadrivalent HPV vaccine (including type 18), ≥99% of persons seroconverted (*197*).

By 6 months postcompletion of a 3-dose (0, 2, and 6 months) schedule among adolescents aged 11–18 years, the proportions with hSBA titers ≥LLOQ were 60% for A22, 89% for A56, 57% for B24, 37% for B44, and 26% for the composite response (*193*). By 12 months postcompletion of any 3-dose series, the proportion with hSBA titers ≥LLOQ was 41%–54% for A22, 69%–76% for A56, 41%–55% for B24, 23%–29% for B44, and 22% for the composite response (*193*,*198*). These proportions remained relatively stable thereafter (at 18, 24, 36, and 48 months postprimary series) (range: 35%–59% for A22, 47%–73% for A56, 41%–57% for B24, 17%–27% for B44, and 16%–19% for the composite response) (*193*,*198*). After a 2-dose schedule, the proportion of recipients with hSBA titers ≥LLOQ was slightly lower than that observed for the 3-dose series but similarly stable 12–48 months after series completion (range: 36%–48% for A22, 54%–60% for A56, 31%–37% for B24, 16%–20% for B44, and, by 48 months, 16% for the composite response) (*193*,*198*).

One month after a booster dose administered 48 months after primary series completion, 94%–98% of persons who received a 2-dose primary series and ≥97% of those who received a 3-dose primary series achieved an hSBA titer ≥1:4 against the four test strains (*19*). Twelve months after MenB-FHbp booster administration, 62%–82% of those who received a 2-dose primary series and 73%–93% of those who received a 3-dose primary series achieved hSBA titers ≥1:4 across the test strains (composite response of 63% for both groups). By 26 months postbooster, further antibody titer waning was observed among persons who received a 2-dose series but not among those who received a 3-dose series: 59%–67% and 71%–90% achieved an hSBA titer ≥1:4 for the four test strains, respectively, although decreases were observed in the composite response for both groups (42% and 46%, respectively).

In addition to these trials demonstrating immunogenicity to the four test strains, several evaluations have assessed immunogenicity against genetically diverse and clinically relevant strains. In one of the clinical trials, 63%–99% of persons vaccinated with a 2-dose primary series at 0 and 2 months and 75%–99% vaccinated with a 3-dose primary series achieved an hSBA titer ≥LLOQ against 10 additional strains (*195*). In a manufacturer-sponsored evaluation, hSBA responses measured against 27 clinically relevant strains (including strains from four U.S. university outbreaks) demonstrated that ≥32% of persons vaccinated with 2 doses and ≥56% vaccinated with 3 doses achieved an hSBA titer ≥1:8 against all test strains (*203*). The proportion of persons who achieved seroprotective hSBA titers was greater for strains expressing the most common FHbp variants in the United States (B24 and A22) at ≥81% and 88%–95%, respectively, after the third dose. In another manufacturer-sponsored study, 1 month after the third MenB-FHbp dose ≥73% of adolescents achieved an hSBA titer ≥1:4 against eight French serogroup B outbreak strains (*204*). In an independent study among vaccinated U.S. health care workers, ≥93% achieved an hSBA titer ≥1:4 against 14 serogroup B strains (including strains from six university outbreaks) by 1 month after the third dose; by 9–11 months postvaccination, 27%–90% remained seroprotected against nine strains tested (*205*).

#### MenB-FHbp Safety

##### Clinical Trials and Research Studies

Among adolescents and young adults, injection site pain (72%–93%), induration (21%–37%), and erythema (10%–24%) were commonly observed local reactions after any dose in either a 2- or 3-dose series (*94*,*194*–*197*,*199*). Headache (27%–67%), fatigue (30%–66%), myalgia (21%–40%), and arthralgia (11%–33%) were the most commonly reported systemic adverse events; fever was reported in 2%–23% of recipients. Reactogenicity did not increase with increasing number of doses. Rates and types of adverse events subsequent to a booster dose administered 4 years after primary series completion were similar to those observed after the primary series (*198*). Most adverse events were mild to moderate, and symptoms typically resolved within 5 days of onset (*94*,*194*–*199*).

Because MenB-FHbp contains factor H binding protein, a theoretical risk exists for development of factor H autoantibodies (implicated in diseases such as atypical hemolytic uremic syndrome and C3 glomerulonephropathy) postvaccination (*206*,*207*); however, whether factor H autoantibodies develop after MenB-FHbp vaccination and, if so, whether they are clinically significant is not known. Among persons who received MenB-FHbp in clinical trials, the proportion with a newly diagnosed autoimmune disease during the trial or during the 6-month follow-up period was low (0.14%) and similar to unvaccinated controls (*208*). Furthermore, the onset of symptoms consistent with the diagnosis occurred before the first vaccination in most of these persons.

##### Postlicensure Safety Monitoring

In a comprehensive review of VAERS reports from licensure in 2014 through June 2018, no new safety concerns were identified (CDC, unpublished data, 2020). A total of 1,719 reports involving MenB-FHbp were identified; among these reports, the median patient age at vaccination was 17 years, and 36% involved simultaneous administration with other vaccines. The most common adverse events reported were fever, headache, and injection site pain. Reported adverse events (e.g., headache, fever, chills, and myalgia) are consistent with those identified in clinical trials. No safety signals related to autoimmune or renal diseases were detected.

After a MenB-FHbp mass vaccination campaign in response to a serogroup B meningococcal disease outbreak on a university campus in which approximately 10,000 doses were administered, adverse events were solicited via survey subsequent to each of the 3 doses (*202*). Among survey respondents, rates of injection site pain, fatigue, myalgia, fevers, and chills were similar to those reported during clinical trials, and no new safety concerns were identified.

### MenB-4C (Bexsero)

MenB-4C was first licensed in the United States in 2015. MenB-4C is licensed for persons aged 10–25 years in the United States; therefore, immunogenicity and safety data for children aged <10 years are not presented in this report. Available data for this age group have been summarized elsewhere (*209*).

Among adolescents and young adults, a 2-dose MenB-4C primary series is immunogenic (*148*,*210*–*213*). Although antibody persistence is difficult to assess because of heterogeneous results by vaccine antigens (FHbp, NhbA, NadA, and PorA) or between studies, different points assessed in different studies, and elevated baseline titers in certain studies, antibody titers appear to wane by 2 years postvaccination (*148*,*213*–*216*). A robust immune response is demonstrated after either a MenB-4C or investigational serogroups A, B, C, W, and Y (MenABCWY) vaccine booster dose administered 2, 4, or 7.5 years after a MenB-4C primary series (*215*,*217*). Although data are limited, persistence after a booster dose likely lasts for several years based on observed and modeled data (*218*). MenB-4C vaccine is safe and well tolerated, although more reactogenic than MenACWY (*188*,*210*–*213*). In clinical trials, the most common local reactions were injection site pain, erythema, and swelling and the most common systemic adverse events were headache, fatigue, and myalgia. In postlicensure safety surveillance, local and systemic adverse events reported are consistent with the clinical trial data (*219*–*221*).

#### MenB-4C Immunogenicity

Clinical trials were conducted to assess immunogenicity to four test strains among persons aged 10–25 years using a 2-dose primary series schedule (0 and 1–2 months), with seroprotection defined as an hSBA titer ≥1:4 or ≥1:5. One month after the second dose, ≥98% of recipients achieved seroprotection against FHbp, ≥97% against NadA, ≥75% against PorA, and ≥68% against NhbA (*148*,*210*–*213*). At 5–6 months after the primary series, ≥82% remained seroprotected against FHbp, ≥93% against NadA, and ≥75% against PorA (NhbA was not assessed) (*148*,*213*). Antibody persistence at further points was variable between studies. In a study conducted among United Kingdom college students, in which the proportion of persons with baseline (prevaccination) titers ≥1:4 ranged from 57% to 69% for FHbp, NadA, and PorA, 85%–97% of vaccinated students remained seroprotected for these antigens at 11 months postcompletion of the primary series (*148*). In a study of Chilean adolescents, in whom baseline titers were also elevated, 75%–93% were seroprotected against FHbp, NadA, and PorA by 18–23 months after the primary series and 29%–84% had seroprotection at 7.5 years (*215*,*216*). However, the proportion seroprotected at 7.5 years was not significantly different from baseline for three of the four antigens in the original study cohort, although higher than an age-matched unvaccinated population for three of four antigens. Among participants in two different clinical trials in which most had low baseline titers, results at 2 years (United States and Poland) and 4 years (Canada and Australia) postvaccination were relatively consistent: 30%–34% seroprotection against FHbp, 84%–94% against NadA, 9%–16% against PorA, and 50%–75% against NhbA (*214*,*215*). In a small clinical trial of adult laboratory workers, in which most participants had high baseline hSBA titers to serogroups A, C, W, and Y and select serogroup B strains, simultaneous administration of MenACWY-CRM and a 3-dose MenB-4C series resulted in robust immune responses through 4 months after the second dose and 1 month after the third dose (*134*).

In extension studies conducted in Chile, Canada, and Australia, a MenB-4C booster was administered either 4 or 7.5 years after completion of the primary series (*215*). One month after booster administration, ≥93% of persons achieved an hSBA titer ≥1:4 across the four vaccine antigens. No data on persistence of the immune response after a booster dose are available for this cohort. However, modeling of the clinical trial data demonstrates that persistence likely lasts for several years (*218*). In an extension of the U.S.-Poland study, 11 persons previously vaccinated with a MenB-4C primary series were randomized to receive an investigational pentavalent (serogroups A, B, C, W, and Y) vaccine, in which the serogroup B component was identical to the components of the licensed MenB-4C product, as a booster dose 2 years postcompletion of the primary series (*217*). One month postbooster, ≥91% of persons achieved an hSBA titer ≥1:5 against FHbp, NadA, and NhbA, and 82% achieved an hSBA titer ≥1:5 against PorA. At 12 months postbooster, all recipients achieved an hSBA titer ≥1:5 against NadA, 82% against FHbp and NhbA, and 45% against PorA, although confidence intervals were wide because of the small study size (*217*).

In addition to clinical trials, several observational immunogenicity studies have been undertaken. After a 2013 mass MenB-4C vaccination campaign at a U.S. university in response to a serogroup B meningococcal disease outbreak caused by a strain predicted by the MATS assay to be covered by both the FHbp and NhbA antigens in MenB-4C, 66% of serosurvey participants achieved an hSBA titer ≥1:4 against the outbreak strain at 2 months after receipt of the 2-dose series; immunogenicity against two vaccine antigen reference strains was high (*222*). Antibody titers against the outbreak strain appeared to wane rapidly postvaccination; by 20 months postvaccination, 24% of recipients remained seroprotected (*223*). Antibody titer waning also was observed among students vaccinated with MenB-4C during a different U.S. university outbreak. The proportion of students with an hSBA titer ≥1:4 against the outbreak strain and three additional university outbreak strains ranged from 53% to 93% 1.5–2 months after completion of the series, and this proportion decreased to 31%–86% at 7 months (*224*). In another evaluation using sera from vaccinated adults, hSBA activity against 18 genetically diverse serogroup B strains (including three reference strains and six university outbreak strains) demonstrated that at 1 month postvaccination, ≥85% of recipients achieved an hSBA titer ≥1:4 against most strains; however, this proportion decreased to 70% for 14 of the strains and 45%–62% for the remaining four strains (two from outbreaks) by 4–6 months postvaccination (*225*).

#### MenB-4C Safety

##### Clinical Trials and Research Studies

Among adolescents and adults, injection site pain (82%–98%), erythema (35%–68%), swelling (26%–47%), and induration (10%–48%) were commonly reported local reactions after primary vaccination (*188*,*210*–*213*). Headache (21%–65%), fatigue and malaise (18%–73%), myalgia (17%–75%), arthralgia (8%–42%), and nausea (8%–35%) were commonly reported systemic adverse events; fever was reported in 1%–10% of recipients. In a clinical trial conducted among laboratory workers in which MenB-4C was administered simultaneously with MenACWY-CRM, local injection site adverse reactions were more common in the arms in which MenB-4C was administered compared with MenACWY-CRM; nausea and headache were more frequently reported when the two vaccines were administered simultaneously compared with MenB-4C administration alone (*134*). After a MenB-4C or investigational MenABCWY booster dose, rates of local or systemic adverse events typically were similar to those observed among persons who received doses as part of primary vaccination (*215*,*217*).

As with MenB-FHbp, MenB-4C contains components that include factor H binding protein. Animal models and an evaluation in humans demonstrated that antibodies generated after MenB-4C vaccination were cross-reactive with human factor H (*226*–*228*). In the human study, a small proportion of persons vaccinated with MenB-4C had transient development of factor H autoantibodies, although factor H function was unaffected (*228*). Although these findings do not suggest that factor H autoantibodies from MenB vaccination are likely to cause factor H–associated autoimmune conditions, the clinical significance remains uncertain, and additional postlicensure safety surveillance will be important. In FDA’s review of MenB-4C clinical trial data, among study participants with an autoimmune disorder diagnosed during the study follow-up period, the onset of symptoms consistent with the disorder occurred before the first vaccination in most trial participants (*70*).

##### Postlicensure Safety Monitoring

MenB-4C safety surveillance was conducted as part of several mass vaccination campaigns in the United States and Canada (*219*–*221*). MenB-4C mass vaccination campaigns were implemented in response to outbreaks at two U.S. universities (approximately 31,000 doses administered), under an expanded access investigational new drug protocol before U.S. licensure of the vaccine, and one university in Canada (approximately 5,000 doses administered) (*220*,*221*). The most commonly reported adverse events were consistent with findings from clinical trial data (e.g., fever, injection site pain, and arm pain), although 0.88 syncopal events per 1,000 persons in the U.S. evaluation were reported. Similarly, safety surveillance for mass vaccination to control increased incidence of serogroup B meningococcal disease in a region of Quebec, Canada, in which nearly 60,000 doses were administered to persons aged ≤20 years, demonstrated local and systemic adverse events consistent with those described in clinical trials, although adverse event–related absenteeism or medical consultations were frequent (*219*). However, four cases of likely idiopathic nephrotic syndrome were identified in vaccinated children aged 2–5 years during the 1-year postvaccination safety monitoring period (*229*). Because of the small number of cases and wide confidence intervals of risk estimates, whether this finding represents a safety signal is unclear.

In a comprehensive review of VAERS reports from licensure in 2015 through June 2018, no new safety concerns were identified (CDC, unpublished data, 2020). A total of 1,470 reports involving MenB-4C vaccination were received; the median patient age was 17 years, and 39% involved simultaneous administration with other vaccines. The most commonly reported adverse events were injection site pain, fever, and headache. Transient decreased mobility of the arm where the vaccine was injected was disproportionately reported for MenB-4C compared with other vaccines. Overall, the reported adverse events were consistent with the findings from clinical trials. No autoimmune or renal disease–related safety signals were detected.

### MenB Vaccine Immunogenicity and Safety in Persons with Underlying Medical Conditions

Immunogenicity of MenB-4C was assessed in children and adolescents aged 2–17 years with certain underlying conditions (*230*). One month postcompletion of a 2-dose primary series, the proportion of persons with complement deficiency or complement inhibitor use with hSBA titers ≥1:5 was 87% for FHbp, 95% for NadA, 68% for PorA, and 73% for NhbA when exogenous complement was used. Among those with asplenia, ≥84% had seroprotection against these four antigens, which was similar to the proportion observed in healthy control participants. However, among complement-deficient persons, when endogenous complement was used, only 41%–68% had seroprotection against the four antigens; among those with terminal component deficiencies or complement inhibitor use, only 17% demonstrated any bactericidal activity postvaccination. In addition, a lack of opsonophagocytic killing of meningococci in the presence of eculizumab in sera from persons vaccinated with MenB-4C has been observed, and cases of serogroup or genogroup B meningococcal disease have been reported despite recent vaccination among persons using eculizumab (*161*,*231*,*232*). Although data are limited for MenB-FHbp, similar concerns exist for the lack of complete protection in vaccinated persons (*69*). Thus, persons with complement deficiency or complement inhibitor use might remain at increased risk for meningococcal disease even if they develop antibodies postvaccination (*69*,*70*,*94*,*201*).

The safety profile of MenB-4C among children and adolescents aged 2–17 years with certain underlying medical conditions was similar to that observed in healthy controls (*230*). In a small Spanish evaluation in adults with complement deficiency, eculizumab use, asplenia, and history of meningococcal disease, plus a microbiologist with an immunodeficiency, the reactogenicity profile of MenB-4C was similar to that reported in clinical trials in adolescents and adults except for a slightly higher rate of fever (13%).

Vaccination in general might activate complement. Thus, patients with complement-mediated diseases, such as those in whom complement inhibitors are used for treatment (e.g., paroxysmal nocturnal hemoglobinuria and atypical hemolytic uremic syndrome), might experience increased signs and symptoms of their underlying disease after vaccination. In a safety review of patients treated with eculizumab in Canada, an increased risk for anemia or hemolysis was observed when patients receiving eculizumab were vaccinated with MenB-4C, particularly in those who received an eculizumab dose within 2 weeks of vaccination (*233*). The Canadian package insert for eculizumab was updated with the manufacturer’s recommendation that patients be vaccinated (with any recommended meningococcal vaccine, not specifically MenB-4C) before or at the same time as eculizumab initiation; those receiving eculizumab treatment are recommended to be vaccinated only when their disease is controlled and the eculizumab concentration in the blood is considered to be high (*234*). No similar safety concerns have been identified in the United States to date; however, meningococcal vaccination is likewise recommended at least 2 weeks before complement inhibitor initiation (*39*,*40*).

### MenB Vaccines in Pregnant Women

Data on safety of MenB vaccines in pregnant women are limited. No controlled trials have been conducted to specifically assess the safety of MenB vaccination among pregnant women and birth outcomes of vaccinated women. Among approximately 6,000 adolescent or adult females enrolled in MenB-FHbp clinical trials, pregnancy was reported during the trial or in the follow-up period in 34 women who had received at least 1 dose (*94*,*194*–*199*,*201*). Among these, four (12%) spontaneous abortions were reported. Among approximately 2,000 adolescent or adult females enrolled in MenB-4C clinical trials, no pregnancies were reported in the published literature (*188*,*210*–*213*,*215*). Evaluation of VAERS through June 2018 identified three reports related to MenB-FHbp vaccination during pregnancy, with no maternal or fetal adverse events reported, and four related to MenB-4C, with one spontaneous abortion reported (CDC, unpublished data, 2020). Both manufacturers maintain pregnancy registries to collect information on birth outcomes after maternal vaccination; however, no data are available (GlaxoSmithKline and Pfizer, personal communications, 2019).

### Effectiveness of MenB Vaccines

After a mass vaccination program among persons aged 2 months–20 years in a region of Canada experiencing increased incidence of serogroup B meningococcal disease due predominantly to a single clone, MenB-4C vaccine effectiveness among all target age groups was estimated at 79% (95% CI: −231% to 99%) in the 4 years postvaccination, although the wide confidence intervals encompassing the null effect value limit the interpretation of the finding (*235*). No additional data on MenB-4C effectiveness are available for adolescents and adults. In the United Kingdom, where infants are vaccinated with MenB-4C at ages 2, 4, and 12 months, vaccine effectiveness among children who completed the series was estimated at 59.1% (95% CI: −31.1% to 87.2%) for all serogroup B strains in the 3 years after vaccination (*236*). No data are available on MenB-FHbp vaccine effectiveness in any age group.

#### Vaccination and Meningococcal Disease Incidence

No information is available on the association between MenB vaccination and meningococcal disease incidence in the United States. This association cannot be assessed because of the low incidence of serogroup B meningococcal disease and low vaccination coverage after the 2015 ACIP recommendation that adolescents be vaccinated on the basis of shared clinical decision-making (22% of adolescents aged 17 years received ≥1 MenB dose in 2019) (*237*).

#### Vaccination and Oropharyngeal Carriage

Current evidence suggests that MenB vaccines probably do not have a substantial effect on the prevalence or acquisition of *N. meningitidis* oropharyngeal carriage. In a large randomized controlled trial in Australia, MenB-4C vaccination of adolescents did not result in a reduction of carriage with *N. meningitidis* serogroup B or other disease-causing *N. meningitidis* serogroups (*238*). In a smaller randomized controlled trial of United Kingdom university students, no immediate reduction in meningococcal carriage was observed in the 1 month after MenB-4C vaccination. By 3 months postvaccination, significantly lower carriage of any meningococcal strain and of capsular groups B, C, W, and Y was observed; however, no specific effect of MenB-4C on serogroup B carriage was observed (*188*). No data from large randomized controlled trials for MenB-FHbp are available. However, two observational carriage evaluations after vaccination of U.S. university students primarily with MenB-FHbp during serogroup B outbreaks demonstrated stable serogroup B carriage rates before and after vaccination, suggesting that MenB-FHbp does not have a large or rapid effect on carriage (*185*,*186*).

### Cost-Effectiveness of MenB Vaccines

Cost-effectiveness of MenB vaccines among U.S. adolescents was first assessed in 2015 and most recently evaluated in 2018 (*10*,*239*). Vaccination strategies included a MenB primary series at age 11 years with a booster at age 16 years, a series at age 16 years, a series at age 18 years, and a series among college students. Cost per QALY saved for these four strategies ranged from $9.6 million to $12.7 million, with the number needed to vaccinate to prevent a case ranging from 152,000 to 305,000 and the number needed to vaccinate to prevent a death ranging from 1.6 million to 2.8 million (*239*).

## Vaccine Administration

MenACWY-D, MenACWY-CRM, MenACWY-TT, MenB-FHbp, and MenB-4C are all administered intramuscularly at a dose of 0.5 mL. However, for MenACWY-CRM, the lyophilized MenA component must be reconstituted with the liquid MenCWY component immediately before administration. If the liquid MenCWY component is inadvertently administered alone without the lyophilized MenA component, revaccination is not necessary for persons who are not planning to travel internationally because serogroup A meningococcal disease is rarely reported in the United States. However, revaccination is necessary if the person plans to travel internationally, particularly to a region where serogroup A meningococcal disease is endemic, or where vaccination is required, such as to the Hajj pilgrimage. In this case, a dose prepared according to the manufacturer’s instructions should be administered as soon as feasible. Additional information on meningococcal vaccine administration is available in the package inserts (*66*–*70*).

## Recommendations for Use of Meningococcal Vaccines

### Adolescents and Young Adults

ACIP recommends routine administration of a MenACWY vaccine for all persons aged 11–18 years (Table 2). In addition, ACIP recommends a MenB vaccine series for persons aged 16–23 years on the basis of shared clinical decision-making to provide short-term protection against most strains of serogroup B meningococcal disease (Table 2). The preferred age for MenB vaccination is 16–18 years.

**TABLE 2:** Recommended meningococcal vaccines and administration schedules for children and adults — Advisory Committee on Immunization Practices, United States, 2020

Age group	Serogroups A, C, W, and Y meningococcal conjugate vaccines MenACWY-D (Menactra, Sanofi Pasteur) *or *MenACWY-CRM (Menveo, GlaxoSmithKline) *or *MenACWY-TT (MenQuadfi, Sanofi Pasteur)	Serogroup B meningococcal vaccines MenB-FHbp (Trumenba, Pfizer) *or *MenB-4C (Bexsero, GlaxoSmithKline)
2 mos–10 yrs	Not routinely recommended See Table 3 for persons at increased risk	No recommendations for use of MenB vaccines in this population*
11–23 yrs	**Primary vaccination^†^:** 1 dose at age 11–12 yrs **Booster:** 1 dose at age 16 yrs if first dose administered before 16th birthday **Catch-up vaccination:** Although routine vaccination is only recommended for adolescents aged 11–18 yrs, MenACWY may be administered to persons aged 19–21 yrs who have not received a dose after their 16th birthday **Note:** MenACWY vaccines are interchangeable	**Primary vaccination:** MenB series at age 16–23 yrs on basis of shared clinical decision-making (preferred age 16–18 yrs) • MenB-FHbp^§^: 2 doses at 0 and 6 mos • MenB-4C: 2 doses ≥1 mo apart **Booster:** Not routinely recommended unless the person becomes at increased risk for meningococcal disease **Note:** MenB-FHbp and MenB-4C are not interchangeable
≥24 yrs	Not routinely recommended See Table 3 for persons at increased risk	Not routinely recommended See Table 3 for persons at increased risk

**Abbreviations:** MenACWY-CRM = meningococcal groups A, C, W, and Y oligosaccharide diphtheria CRM_197_ conjugate vaccine; MenACWY-D = meningococcal groups A, C, W, and Y polysaccharide diphtheria toxoid conjugate vaccine; MenACWY-TT = meningococcal groups A, C, W, and Y polysaccharide tetanus toxoid conjugate vaccine; MenB-4C = four-component meningococcal group B vaccine; MenB-FHbp = meningococcal group B factor H binding protein vaccine.

* MenB vaccines are licensed in the United States only for persons aged 10–25 years.

^†^ College freshmen living in residence halls should receive at least 1 dose of MenACWY within 5 years before college entry. The preferred timing of the most recent dose is on or after their 16th birthday. If only 1 dose of vaccine was administered before the 16th birthday, a booster dose should be administered before enrollment. Adolescents who received a first dose after their 16th birthday do not need another dose before college entry unless it has been more than 5 years since the dose. Certain schools, colleges, and universities have policies requiring vaccination against meningococcal disease as a condition of enrollment.

^§^ When given to healthy adolescents who are not otherwise at increased risk for meningococcal disease, 2 doses of MenB-FHbp should be administered at 0 and 6 months. For persons at increased risk for meningococcal disease and for use during serogroup B meningococcal disease outbreaks, 3 doses of MenB-FHbp should be administered at 0, 1–2, and 6 months to provide earlier protection and maximize short-term immunogenicity.

#### MenACWY Vaccines

ACIP recommends a single dose of MenACWY at age 11 or 12 years followed by a booster dose administered at age 16 years (Table 2). Children who received MenACWY at age 10 years do not need an additional dose at age 11–12 years but should receive the booster dose at age 16 years. Children who received MenACWY before age 10 years and with no ongoing risk for meningococcal disease for which boosters are recommended should still receive MenACWY according to the recommended adolescent schedule, with the first dose at age 11–12 years and a booster dose at age 16 years. For example, a healthy child who received MenACWY at age 9 years because of short-term travel to a country where meningococcal disease is hyperendemic or epidemic and who is not otherwise at increased risk should receive the MenACWY at age 11–12 years according to the recommended ACIP adolescent vaccination schedule. Children who received MenACWY before age 10 years and for whom boosters are recommended because of an ongoing increased risk for meningococcal disease (e.g., those with complement deficiency, HIV infection, or asplenia) should follow the booster schedule for persons at increased risk.

Adolescents who receive their first dose at age 13–15 years should receive a booster dose at age 16–18 years; the booster dose can be administered at any time, as long as a minimum interval of 8 weeks between doses is maintained. Adolescents who receive a first dose after their 16th birthday do not need a booster dose unless they become at increased risk for meningococcal disease. Persons aged 19–21 years who have not received a dose after their 16th birthday can receive a single MenACWY dose as part of catch-up vaccination. MenACWY vaccines are interchangeable; the same vaccine product is recommended, but not required, for all doses. MenACWY vaccines can be administered simultaneously with other vaccines indicated for this age group, but at a different anatomic site, if feasible. MenACWY-TT, which is conjugated to tetanus toxoid, is only licensed for the prevention of meningococcal disease; use of this vaccine does not replace doses or affect the dosing intervals of routinely recommended tetanus toxoid–containing vaccines in any age group.

#### MenB Vaccines

MenB vaccination is not routinely recommended for all adolescents. Instead, ACIP recommends a MenB series for persons aged 16–23 years (preferred age 16–18 years) on the basis of shared clinical decision-making (*240*) (Table 2). Shared clinical decision-making refers to an individually based vaccine recommendation informed by a decision-making process between the health care provider and the patient or parent/guardian. Considerations for shared clinical decision-making for vaccine administration and timing of administration might include

- the serious nature of meningococcal infections, with high rates of death and permanent sequelae in those who develop invasive disease;
- the low number of serogroup B meningococcal disease cases (average of 34 serogroup B cases annually among persons aged 16–23 years in the United States during 2015–2018);
- the increased risk among college students, especially those who are freshmen, attend a 4-year university, live in on-campus housing, or participate in sororities and fraternities;
- the protection provided by MenB vaccines against most strains of serogroup B *N. meningitidis;*
- the estimated relatively short duration of MenB protection (antibody waning within 1–2 years postcompletion of the primary series); and
- the evidence to date suggesting that MenB vaccination has no effect on meningococcal carriage (i.e., MenB vaccines might provide individual protection against serogroup B disease but herd protection is unlikely).

For adolescents who are not otherwise at increased risk for meningococcal disease (e.g., due to complement deficiency or asplenia), a 2-dose series of MenB vaccine should be administered as follows: 2 doses of MenB-FHbp administered at 0 and 6 months or 2 doses of MenB-4C administered at 0 and ≥1 month. If the second dose of MenB-FHbp is administered earlier than 6 months after the first dose, a third dose should be administered at least 4 months after the second dose. Either of the MenB vaccines can be used when indicated; ACIP does not state a product preference. However, MenB vaccines are not interchangeable, and the same vaccine product must be used for all doses. If one MenB dose was received but the vaccine product is unknown, the series must be restarted with either product to ensure completion of a 2-dose series using the same product. If 2 doses were administered using different MenB products, one product should be selected for administration of an additional dose at an appropriate interval to ensure valid completion of a MenB series; the dose from the product not selected for series completion should be considered invalid. For situations in which a MenB dose or doses must be repeated, a minimum interval of 4 weeks should be used between any 2 doses. MenB vaccines can be administered simultaneously with other vaccines indicated for this age group, but at a different anatomic site, if feasible.

### Persons at Increased Risk for Meningococcal Disease

Persons at increased risk for meningococcal disease are recommended to receive routine meningococcal vaccination. Vaccine product, number of doses, and booster dose recommendations are based on age and risk factors (Tables 3, 4, 5, 6, 7, 8, 9, and 10). Although evidence suggests that vaccination might not adequately prevent meningococcal infections among persons with certain complement deficiencies or those using a complement inhibitor (*66*–*70*), these persons should continue to be vaccinated according to recommendations because of a possible benefit among persons at high risk for infection (Table 4). Persons using complement inhibitor should be vaccinated at least 2 weeks before complement inhibitor initiation unless the risks for delaying treatment outweigh the risks for developing meningococcal disease. Among unvaccinated patients for whom complement inhibitor therapy cannot be delayed, antimicrobial prophylaxis (e.g., penicillin) should be administered alongside meningococcal vaccination and continued for 2 weeks after vaccine administration (*39*,*40*). In addition, providers might consider antimicrobial prophylaxis for the duration of complement inhibitor therapy. Among persons undergoing elective splenectomy, meningococcal vaccines should be administered at least 2 weeks before surgery, if possible; otherwise, they should be administered after the procedure as soon as the patient’s condition is stable (*241*).

**TABLE 3:** Recommended meningococcal vaccines for persons at increased risk for meningococcal disease — Advisory Committee on Immunization Practices, United States, 2020

Risk group	MenACWY vaccine	MenB vaccine	Table
Persons with complement component deficiency (e.g., C5–C9, properdin, factor H, or factor D), including patients using a complement inhibitor	Aged ≥2 mos	Aged ≥10 yrs	4
Persons with functional or anatomic asplenia (including sickle cell disease)	Aged ≥2 mos	Aged ≥10 yrs	5
Persons with HIV infection	Aged ≥2 mos	No recommendation	6
Microbiologists routinely exposed to *Neisseria meningitidis*	Age appropriate*	Age appropriate^†^	7
Persons exposed during an outbreak of meningococcal disease due to a vaccine-preventable serogroup	Aged ≥2 mos	Aged ≥10 yrs	8
Persons who travel to or live in countries where meningococcal disease is hyperendemic or epidemic	Aged ≥2 mos	No recommendation	9
College freshmen living in residence halls	Age appropriate*	No recommendation	10
Military recruits	Age appropriate*	No recommendation	10

**Abbreviations:** HIV = human immunodeficiency virus; MenACWY = meningococcal groups A, C, W, and Y; MenB = meningococcal group B.

* Persons aged ≥2 months in these risk groups are recommended to receive MenACWY vaccination.

^†^ Persons aged ≥10 years in this risk group are recommended to receive MenB vaccination.

**TABLE 4:** Recommended vaccination schedule and intervals for persons with persistent complement deficiencies* (including patients using a complement inhibitor)^†^ — Advisory Committee on Immunization Practices, United States, 2020

Age group	Serogroups A, C, W, and Y meningococcal conjugate vaccines MenACWY-D (Menactra, Sanofi Pasteur)^§^ *or *MenACWY-CRM (Menveo, GlaxoSmithKline)^¶^ *or* MenACWY-TT (MenQuadfi, Sanofi Pasteur)**	Serogroup B meningococcal vaccines MenB-FHbp (Trumenba, Pfizer) *or *MenB-4C (Bexsero, GlaxoSmithKline)
2–23 mos	**Primary vaccination:** MenACWY-D (aged ≥9 mos): 2 doses ≥12 wks apart ***or*** MenACWY-CRM if first dose at age • 2 mos: 4 doses at 2, 4, 6, and 12 mos • 3–6 mos: See catch-up schedule^§§^ • 7–23 mos: 2 doses (second dose ≥12 wks after the first dose and after the 1st birthday)	No recommendations for use of MenB vaccines in this population^††^
2–9 yrs	**Primary vaccination^¶¶^:** MenACWY-D*** ***or*** MenACWY-CRM ***or*** MenACWY-TT: 2 doses ≥8 wks apart **Boosters (if person remains at increased risk)^†††^:** • Aged <7 yrs: Single dose at 3 yrs after primary vaccination and every 5 yrs thereafter • Aged ≥7 yrs: Single dose at 5 yrs after primary vaccination and every 5 yrs thereafter	No recommendations for use of MenB vaccines in this population^††^
≥10 yrs	**Primary vaccination^††^:** MenACWY-D ***or*** MenACWY-CRM ***or*** MenACWY-TT: 2 doses ≥8 wks apart **Boosters (if person remains at increased risk)^†††^:** Single dose at 5 yrs after primary vaccination and every 5 yrs thereafter	**Primary vaccination^††^:** MenB-FHbp: 3 doses at 0, 1–2, and 6 mos ***or*** MenB-4C: 2 doses ≥1 mo apart **Boosters (if person remains at increased risk)^§§§^:** Single dose at 1 yr after completion of primary vaccination and every 2–3 yrs thereafter **Note:** MenB-FHbp and MenB-4C are not interchangeable

**Abbreviations:** DTaP = diphtheria and tetanus toxoids and acellular pertussis vaccine; MenACWY-CRM = meningococcal groups A, C, W, and Y oligosaccharide diphtheria CRM_197_ conjugate vaccine; MenACWY-D = meningococcal groups A, C, W, and Y polysaccharide diphtheria toxoid conjugate vaccine; MenACWY-TT = meningococcal groups A, C, W, and Y polysaccharide tetanus toxoid conjugate vaccine; MenB-4C = four-component meningococcal group B vaccine; MenB-FHbp = meningococcal group B factor H binding protein vaccine.

* Persistent complement deficiencies include C3, C5–C9, properdin, factor H, or factor D.

^†^ Includes eculizumab (Soliris) and ravulizumab (Ultomiris). Meningococcal vaccines should be administered at least 2 weeks before the first dose of complement inhibitor, unless the risk for delaying complement therapy outweighs the risk for developing meningococcal disease.

^§^ Licensed in the United States only for persons aged 9 months–55 years. Vaccination of persons aged ≥56 years is considered off-label.

^¶^ Licensed in the United States only for persons aged 2 months–55 years. Vaccination of persons aged ≥56 years is considered off-label.

** Licensed in the United States only for persons aged ≥2 years.

^††^ Licensed in the United States only for persons aged 10–25 years. Vaccination of persons aged ≥26 years is considered off-label.

^§§^ If MenACWY-CRM is initiated at ages 3–6 months, catch-up vaccination includes doses at intervals of 8 weeks until the infant is aged ≥7 months, at which time an additional dose is administered at age ≥7 months, followed by a dose at least 12 weeks later and after the 1st birthday.

^¶¶^ Primary vaccination licensed as a single dose in persons aged 2–55 years for MenACWY-D and MenACWY-CRM or ≥2 years for MenACWY-TT. Two-dose primary series is considered off-label.

*** MenACWY-D should be given either before or at the same time as DTaP to avoid interference with the immune response to meningococcal vaccine in children.

^†††^ Licensed in the United States only for a single booster dose for persons aged 15–55 years for MenACWY-D and MenACWY-CRM or aged ≥15 years for MenACWY-TT. Booster doses administered outside of these ages or administration of >1 booster dose are considered off-label.

^§§§^ Licensed in the United States only for a primary series. Administration of booster doses is considered off-label.

**TABLE 5:** Recommended vaccination schedule and intervals for persons with anatomic and functional asplenia (including sickle cell disease) — Advisory Committee on Immunization Practices, United States, 2020

Age group	Serogroups A, C, W, and Y meningococcal conjugate vaccines MenACWY-D (Menactra, Sanofi Pasteur)* *or *MenACWY-CRM (Menveo, GlaxoSmithKline)^†^ *or *MenACWY-TT (MenQuadfi, Sanofi Pasteur)^§^	Serogroup B meningococcal vaccines MenB-FHbp (Trumenba, Pfizer) *or *MenB-4C (Bexsero, GlaxoSmithKline)
2–23 mos	**Primary vaccination:** MenACWY-CRM: If first dose at age • 2 mos: 4 doses at 2, 4, 6, and 12 mos • 3–6 mos: See catch-up schedule^¶^ • 7–23 mos: 2 doses (second dose ≥12 wks after the first dose and after the 1st birthday)	No recommendations for use of MenB vaccines in this population**
2–9 yrs	**Primary vaccination^††^:** MenACWY-D^§§,¶¶^: 2 doses ≥8 wks apart and ≥4 wks after completion of PCV13 series ***or*** MenACWY-CRM ***or*** MenACWY-TT: 2 doses ≥8 wks apart Boosters (if person remains at increased risk)***: • Aged <7 yrs: Single dose at 3 yrs after vaccination and every 5 yrs thereafter • Aged ≥7 yrs: Single dose at 5 yrs and every 5 yrs thereafter	No recommendations for use of MenB vaccines in this population**
≥10 yrs	**Primary vaccination^††^:** MenACWY-D^¶¶^: 2 doses ≥8 wks apart and ≥4 wks after completion of PCV13 series ***or*** MenACWY-CRM ***or*** MenACWY-TT: 2 doses ≥8 wks apart Boosters (if person remains at increased risk)***: Single dose at 5 yrs after primary vaccination and every 5 yrs thereafter	Primary vaccination**: MenB-FHbp: 3 doses at 0, 1–2, and 6 mos ***or*** MenB-4C: 2 doses ≥1 mo apart **Boosters (if person remains at increased risk)^†††^:** Single dose at 1 yr after completion of primary vaccination and every 2–3 yrs thereafter **Note:** MenB-FHbp and MenB-4C are not interchangeable

**Abbreviations:** DTaP = diphtheria and tetanus toxoids and acellular pertussis vaccine; MenACWY-CRM = meningococcal groups A, C, W, and Y oligosaccharide diphtheria CRM_197_ conjugate vaccine; MenACWY-D = meningococcal groups A, C, W, and Y polysaccharide diphtheria toxoid conjugate vaccine; MenACWY-TT = meningococcal groups A, C, W, and Y polysaccharide tetanus toxoid conjugate vaccine; MenB-4C = four-component meningococcal group B vaccine; MenB-FHbp = meningococcal group B factor H binding protein vaccine; PCV = pneumococcal conjugate vaccine.

* Licensed in the United States only for persons aged 9 months–55 years. Vaccination of persons aged ≥56 years is considered off-label.

^†^ Licensed in the United States only for persons aged 2 months–55 years. Vaccination of persons aged ≥56 years is considered off-label.

^§^ Licensed in the United States only for persons aged ≥2 years.

^¶^ If MenACWY-CRM is initiated at ages 3–6 months, catch-up vaccination includes doses at intervals of 8 weeks until the infant is aged ≥7 months, at which time an additional dose is administered at age ≥7 months, followed by a dose at least 12 weeks later and after the 1st birthday.

** Licensed in the United States only for persons aged 10–25 years. Vaccination of persons aged ≥26 years is considered off-label.

^††^ Primary vaccination licensed as a single dose in persons aged 2–55 years for MenACWY-D and MenACWY-CRM or ≥2 years for MenACWY-TT. Two-dose primary series is considered off-label.

^§§^ MenACWY-D should be given either before or at the same time as DTaP to avoid interference with the immune response to meningococcal vaccine in children.

^¶¶^ Because of the high risk for invasive pneumococcal disease, children with functional or anatomic asplenia or human immunodeficiency virus infection should not be vaccinated with MenACWY-D (Menactra) before age 2 years to avoid interference with the immune response to PCV. If MenACWY-D is used in a person (of any age) with these conditions, it should not be administered until at least 4 weeks after completion of all PCV doses.

*** Licensed in the United States only for a single booster dose for persons aged 15–55 years for MenACWY-D and MenACWY-CRM or aged ≥15 years for MenACWY-TT. Booster doses administered outside of these ages or administration of >1 booster dose are considered off-label.

^†††^ Licensed in the United States only for a primary series. Administration of booster doses is considered off-label.

**TABLE 6:** Recommended vaccination schedule and intervals for persons with human immunodeficiency virus infection — Advisory Committee on Immunization Practices, United States, 2020

Age group	Serogroups A, C, W, and Y meningococcal conjugate vaccines MenACWY-D (Menactra, Sanofi Pasteur)* *or* MenACWY-CRM (Menveo, GlaxoSmithKline)^†^ *or *MenACWY-TT (MenQuadfi, Sanofi Pasteur)^§^	Serogroup B meningococcal vaccines MenB-FHbp (Trumenba, Pfizer) *or* MenB-4C (Bexsero, GlaxoSmithKline)
2–23 mos	**Primary vaccination:** MenACWY-CRM: If first dose at age • 2 mos: 4 doses at 2, 4, 6, and 12 mos • 3–6 mos: See catch-up schedule^¶^ • 7–23 mos: 2 doses (second dose ≥12 wks after the first dose and after the 1st birthday)	No recommendations for use of MenB vaccines in these populations unless otherwise indicated (in persons aged ≥10 yrs)
≥2 yrs	Primary vaccination**: MenACWY-D^††,§§^: 2 doses ≥8 wks apart and ≥4 wks after completion of PCV13 series ***or*** MenACWY-CRM ***or*** MenACWY-TT: 2 doses ≥8 wks apart **Boosters (if person remains at increased risk)^¶¶^:** • Aged <7 yrs: Single dose at 3 yrs after primary vaccination and every 5 yrs thereafter • Aged ≥7 yrs: Single dose at 5 yrs after primary vaccination and every 5 yrs thereafter	See Table 2 for recommendations in persons aged 16–23 yrs

**Abbreviations:** DTaP = diphtheria and tetanus toxoids and acellular pertussis vaccine; MenACWY-CRM = meningococcal groups A, C, W, and Y oligosaccharide diphtheria CRM_197_ conjugate vaccine; MenACWY-D = meningococcal groups A, C, W, and Y polysaccharide diphtheria toxoid conjugate vaccine; MenACWY-TT = meningococcal groups A, C, W, and Y polysaccharide tetanus toxoid conjugate vaccine; MenB-4C = four-component meningococcal group B vaccine; MenB-FHbp = meningococcal group B factor H binding protein vaccine; PCV = pneumococcal conjugate vaccine; Td = tetanus and diphtheria toxoids vaccine; Tdap = tetanus toxoid, reduced diphtheria toxoid, and acellular pertussis vaccine.

* Licensed in the United States only for persons aged 9 months–55 years. Vaccination of persons aged ≥56 years is considered off-label.

^†^ Licensed in the United States only for persons aged 2 months–55 years. Vaccination of persons aged ≥56 years is considered off-label.

^§^ Licensed in the United States only for persons aged ≥2 years.

^¶^ If MenACWY-CRM is initiated at ages 3–6 months, catch-up vaccination includes doses at intervals of 8 weeks until the infant is aged ≥7 months, at which time an additional dose is administered at age ≥7 months, followed by a dose at least 12 weeks later and after the 1st birthday.

** Primary vaccination licensed as a single dose in persons aged 2–55 years for MenACWY-D and MenACWY-CRM or ≥2 years for MenACWY-TT. Two-dose primary series is considered off-label.

^††^ MenACWY-D should be given either before or at the same time as DTaP to avoid interference with the immune response to meningococcal vaccine in children. MenACWY-D may be given at any time in relation to Tdap or Td.

^§§^ Because of the high risk for invasive pneumococcal disease, children with functional or anatomic asplenia or human immunodeficiency virus infection should not be vaccinated with MenACWY-D (Menactra) before age 2 years to avoid interference with the immune response to PCV. If MenACWY-D is used in a person (of any age) with functional or anatomic asplenia or HIV infection, it should not be administered until at least 4 weeks after completion of all PCV doses.

^¶¶^ Licensed in the United States only for a single booster dose for persons aged 15–55 years for MenACWY-D and MenACWY-CRM or aged ≥15 years for MenACWY-TT. Booster doses administered outside of these ages or administration of >1 booster dose are considered off-label.

**TABLE 7:** Recommended vaccination schedule and intervals for microbiologists routinely exposed to isolates of *Neisseria meningitidis* — Advisory Committee on Immunization Practices, United States, 2020

Age group	Serogroups A, C, W, and Y meningococcal conjugate vaccines MenACWY-D (Menactra, Sanofi Pasteur)* *or *MenACWY-CRM (Menveo, GlaxoSmithKline)^†^ *or *MenACWY-TT (MenQuadfi, Sanofi Pasteur)^§^	Serogroup B meningococcal vaccines MenB-FHbp (Trumenba, Pfizer) *or *MenB-4C (Bexsero, GlaxoSmithKline)
≥10 yrs	**Primary vaccination:** MenACWY-D ***or*** MenACWY-CRM ***or*** MenACWY-TT: 1 dose Boosters (if person remains at increased risk)**: Single dose at 5 yrs after primary vaccination and every 5 yrs thereafter	**Primary vaccination^¶^:** MenB-FHbp: 3 doses at 0, 1–2, and 6 mos ***or*** MenB-4C: 2 doses ≥1 mo apart **Boosters (if person remains at increased risk)^††^:** Single dose at 1 yr after completion of primary vaccination and every 2–3 yrs thereafter **Note:** MenB-FHbp and MenB-4C are not interchangeable

**Abbreviations:** MenACWY-CRM = meningococcal groups A, C, W, and Y oligosaccharide diphtheria CRM_197_ conjugate vaccine; MenACWY-D = meningococcal groups A, C, W, and Y polysaccharide diphtheria toxoid conjugate vaccine; MenACWY-TT = meningococcal groups A, C, W, and Y polysaccharide tetanus toxoid conjugate vaccine; MenB-4C = four-component meningococcal group B vaccine; MenB-FHbp = meningococcal group B factor H binding protein vaccine.

* Licensed in the United States only for persons aged 9 months–55 years. Vaccination of persons aged ≥56 years is considered off-label.

^†^ Licensed in the United States only for persons aged 2 months–55 years. Vaccination of persons aged ≥56 years is considered off-label.

^§^ Licensed in the United States only for persons aged ≥2 years.

^¶^ Licensed in the United States only for persons aged 10–25 years. Vaccination of persons aged ≥26 years is considered off-label.

** Licensed in the United States only for a single booster dose for persons aged 15–55 years for MenACWY-D and MenACWY-CRM or aged ≥15 years for MenACWY-TT. Booster doses administered outside of these ages or administration of >1 booster dose are considered off-label.

^††^ Licensed in the United States only for a primary series. Administration of booster doses is considered off-label.

**TABLE 8:** Recommended vaccination schedule and intervals for persons who are at risk during an outbreak* attributable to a vaccine serogroup — Advisory Committee on Immunization Practices, United States, 2020

Age group	Serogroups A, C, W, and Y meningococcal conjugate vaccines MenACWY-D (Menactra, Sanofi Pasteur)^†^ *or *MenACWY-CRM (Menveo, GlaxoSmithKline)^§^ *or *MenACWY-TT (MenQuadfi, Sanofi Pasteur)^¶^	Serogroup B meningococcal vaccines MenB-FHbp (Trumenba, Pfizer) *or* MenB-4C (Bexsero, GlaxoSmithKline)
2–23 mos	**Primary vaccination: **MenACWY-D (aged ≥9 mos): 2 doses ≥12 wks apart ***or*** MenACWY-CRM: If first dose at age • 2 mos: 4 doses at 2, 4, 6, and 12 mos • 3–6 mos: See catch-up schedule^††^ • 7–23 mos: 2 doses (second dose ≥12 wks after the first dose and after the 1st birthday)	No recommendations for use of MenB vaccines in this population**
2–9 yrs	**Primary vaccination:** MenACWY-D^§§ ^***or*** MenACWY-CRM ***or*** MenACWY-TT: 1 dose **Boosters (if previously vaccinated and identified as being at increased risk)^¶¶^:** • Aged <7 yrs: Single dose if ≥3 yrs since vaccination • Aged ≥7 yrs: single dose if ≥5 yrs since vaccination	No recommendations for use of MenB vaccines in this population**
≥10 yrs	**Primary vaccination:** MenACWY-D ***or*** MenACWY-CRM ***or*** MenACWY-TT: 1 dose **Boosters (if person previously vaccinated and identified as being at increased risk during an outbreak)^¶¶^:** • Aged <7 yrs: Single dose if ≥3 yrs since vaccination • Aged ≥7 yrs: Single dose if ≥5 yrs since vaccination	**Primary vaccination:** MenB-FHbp: 3 doses at 0, 1–2, and 6 mos ***or*** MenB-4C: 2 doses ≥1 mo apart Boosters (if person previously vaccinated and identified as being at increased risk during an outbreak)***: Single dose if ≥1 yr after MenB primary series completion (≥6 mos interval might also be considered by public health professionals) **Note:** MenB-FHbp and MenB-4C are not interchangeable

**Abbreviations:** DTaP = diphtheria and tetanus toxoids and acellular pertussis vaccine; MenACWY-CRM = meningococcal groups A, C, W, and Y oligosaccharide diphtheria CRM_197_ conjugate vaccine; MenACWY-D = meningococcal groups A, C, W, and Y polysaccharide diphtheria toxoid conjugate vaccine; MenACWY-TT = meningococcal groups A, C, W, and Y polysaccharide tetanus toxoid conjugate vaccine; MenB-4C = four-component meningococcal group B vaccine; MenB-FHbp = meningococcal group B factor H binding protein vaccine.

* Detailed recommendations on outbreak management are available at https://www.cdc.gov/meningococcal/downloads/meningococcal-outbreak-guidance.pdf.

^†^ Licensed in the United States only for persons aged 9 months–55 years. Vaccination of persons aged ≥56 years is considered off-label.

^§^ Licensed in the United States only for persons aged 2 months–55 years. Vaccination of persons aged ≥56 years is considered off-label.

^¶^ Licensed in the United States only for persons aged ≥2 years.

** Licensed in the United States only for persons aged 10–25 years. Vaccination of persons aged ≥26 years is considered off-label.

^††^ If MenACWY-CRM is initiated at ages 3–6 months, catch-up vaccination includes doses at intervals of 8 weeks until the infant is aged ≥7 months, at which time an additional dose is administered at age ≥7 months, followed by a dose at least 12 weeks later and after the 1st birthday.

^§§^ MenACWY-D should be given either before or at the same time as DTaP to avoid interference with the immune response to meningococcal vaccine in children.

^¶¶^ Licensed in the United States only for a single booster dose for persons aged 15–55 years for MenACWY-D and MenACWY-CRM or aged ≥15 years for MenACWY-TT. Booster doses administered outside of these ages or administration of >1 booster dose are considered off-label.

*** Licensed in the United States only for a primary series. Administration of booster doses is considered off-label.

**TABLE 9:** Recommended vaccination schedule and intervals for persons who travel to or are residents of countries where meningococcal disease is hyperendemic or epidemic* — Advisory Committee on Immunization Practices, United States, 2020

Age group	Serogroups A, C, W, and Y meningococcal conjugate vaccines MenACWY-D (Menactra, Sanofi Pasteur)^†^ *or *MenACWY-CRM (Menveo, GlaxoSmithKline)^§^ *or *MenACWY-TT (MenQuadfi, Sanofi Pasteur)^¶^	Serogroup B meningococcal vaccines MenB-FHbp (Trumenba, Pfizer) *or *MenB-4C (Bexsero, GlaxoSmithKline)
2–23 mos	**Primary vaccination:** MenACWY-D (aged ≥9 mos)**: 2 doses ≥12 wks apart (may be administered as early as ≥8 wks apart in travelers) ***or*** MenACWY-CRM: If first dose at age • 2 mos: 4 doses at 2, 4, 6, and 12 mos • 3–6 mos: See catch-up schedule^§§^ • 7–23 mos: 2 doses (second dose ≥12 wks after the first dose and after the 1st birthday)	No recommendations for use of MenB vaccines in this population unless otherwise indicated^††^
≥2 yrs	**Primary vaccination:** MenACWY-D** ***or*** MenACWY-CRM ***or*** MenACWY-TT: 1 dose Boosters (if person remains at increased risk)^¶¶,^*** • Aged <7 yrs: Single dose at 3 yrs after primary vaccination and every 5 yrs thereafter • Aged ≥7 yrs: Single dose at 5 yrs after primary vaccination and every 5 yrs thereafter	See Table 2 for recommendations in persons aged 16**–**23 yrs

**Abbreviations:** DTaP = diphtheria and tetanus toxoids and acellular pertussis vaccine; MenACWY-CRM = meningococcal groups A, C, W, and Y oligosaccharide diphtheria CRM_197_ conjugate vaccine; MenACWY-D = meningococcal groups A, C, W, and Y polysaccharide diphtheria toxoid conjugate vaccine; MenACWY-TT = meningococcal groups A, C, W, and Y polysaccharide tetanus toxoid conjugate vaccine; MenB-4C = four-component meningococcal group B vaccine; MenB-FHbp = meningococcal group B factor H binding protein vaccine; Td = tetanus and diphtheria toxoids vaccine; Tdap = tetanus toxoid, reduced diphtheria toxoid, and acellular pertussis vaccine.

* For international travelers, vaccination is recommended for those visiting the parts of sub-Saharan Africa known as the meningitis belt during the dry season (December–June). Vaccination may also be considered for travelers to countries that contain areas included in the meningitis belt but who travel to areas outside of the meningitis belt zone. Advisories for travelers to other countries are issued by CDC when epidemics of meningococcal disease caused by vaccine-preventable serogroups are detected. Traveler’s health information is available from CDC toll free by calling 1-877-394-8747 (1-877-FYI-TRIP) or at https://wwwnc.cdc.gov/travel. Additional information about geographic areas for which vaccination is recommended can be obtained from international health clinics for travelers and state health departments.

^†^ Licensed in the United States only for persons aged 9 months–55 years. Vaccination of persons aged ≥56 years is considered off-label.

^§^ Licensed in the United States only for persons aged 2 months–55 years. Vaccination of persons aged ≥56 years is considered off-label.

^¶^ Licensed in the United States only for persons aged ≥2 years.

** MenACWY-D should be given either before or at the same time as DTaP to avoid interference with the immune response to meningococcal vaccine in children. MenACWY-D may be given at any time in relation to Tdap or Td.

^††^ Some countries recommend routine use of MenB vaccines for infants; persons living in these countries might follow the vaccination recommendations of these countries.

^§§^ If MenACWY-CRM is initiated at ages 3–6 months, catch-up vaccination includes doses at intervals of 8 weeks until the infant is aged ≥7 months, at which time an additional dose is administered at age ≥7 months, followed by a dose at least 12 weeks later and after the 1st birthday.

^¶¶^ Licensed in the United States only for a single booster dose for persons aged 15–55 years for MenACWY-D and MenACWY-CRM or aged ≥15 years for MenACWY-TT. Booster doses administered outside of these ages or administration of >1 booster dose are considered off-label.

*** International travelers should receive a booster dose of MenACWY if the last dose was administered 3–5 or more years previously (depending on the age at most recent dose received). Vaccination is required by the Kingdom of Saudi Arabia (KSA) for all travelers to Mecca during the Hajj and Umrah pilgrimages. Travelers should confirm current vaccination requirements with the KSA embassy.

**TABLE 10:** Recommended vaccination schedule and intervals for college freshmen living in residence halls* and military recruits — Advisory Committee on Immunization Practices, United States, 2020

Age group	Serogroups A, C, W, and Y meningococcal conjugate vaccines MenACWY-D (Menactra, Sanofi Pasteur)^†^ *or *MenACWY-CRM (Menveo, GlaxoSmithKline)^§^ *or *MenACWY-TT (MenQuadfi, Sanofi Pasteur)^¶^	Serogroup B meningococcal vaccines MenB-FHbp (Trumenba, Pfizer) *or *MenB-4C (Bexsero, GlaxoSmithKline)
≥10 yrs	**Primary vaccination:** MenACWY-D ***or*** MenACWY-CRM ***or*** MenACWY-TT: 1 dose Boosters**: • College freshmen living in residence halls: Not routinely recommended unless person becomes at increased risk due to another indication • Military recruits: Every 5 yrs on basis of assignment^††^	No recommendations for use of MenB vaccines in this population unless otherwise indicated See Table 2 for recommendations in persons aged 16–23 yrs

**Abbreviations:** MenACWY-CRM = meningococcal groups A, C, W, and Y oligosaccharide diphtheria CRM_197_ conjugate vaccine; MenACWY-D = meningococcal groups A, C, W, and Y polysaccharide diphtheria toxoid conjugate vaccine; MenACWY-TT = meningococcal groups A, C, W, and Y polysaccharide tetanus toxoid conjugate vaccine; MenB-4C = four-component meningococcal group B vaccine; MenB-FHbp = meningococcal group B factor H binding protein vaccine.

* College freshmen living in residence halls should receive at least 1 dose of MenACWY within 5 years before college entry. The preferred timing of the most recent dose is on or after their 16th birthday. If only 1 dose of vaccine was administered before the 16th birthday, a booster dose should be administered before enrollment. Adolescents who received a first dose after their 16th birthday do not need another dose before college entry unless it has been more than 5 years since the dose. Some schools, colleges, and universities have policies requiring vaccination against meningococcal disease as a condition of enrollment.

^†^ Licensed in the United States only for persons aged 9 months–55 years. Vaccination of persons aged ≥56 years is considered off-label.

^§^ Licensed in the United States only for persons aged 2 months–55 years. Vaccination of persons aged ≥56 years is considered off-label.

^¶^ Licensed in the United States only for persons aged ≥2 years.

^**^ Licensed in the United States only for a single booster dose for persons aged 15–55 years for MenACWY-D and MenACWY-CRM or aged ≥15 years for MenACWY-TT. Booster doses administered outside of these ages or administration of >1 booster dose are considered off-label.

^††^ Vaccination recommendations for military personnel are made by the U.S. Department of Defense on the basis of high-risk travel requirements.

#### MenACWY Vaccines

Children at increased risk for meningococcal disease caused by serogroups A, C, W, or Y (Box 1) who received MenACWY at age <11 years and for whom booster vaccination is recommended because of an ongoing increased risk should follow the booster dose schedule (Tables 4, 5, 6, 7, 8, and 9), not the routine adolescent schedule. For example, a child with HIV infection who received MenACWY at age 9 years should receive the next dose at age 14 years. Booster doses administered to children aged <15 years, repeated booster doses, and booster doses administered at an interval of <4 years are not licensed in the United States and are considered off-label (Table 11).

**TABLE 11:** Off-label meningococcal vaccination recommendations for persons at increased risk for meningococcal disease, by age group and indication — Advisory Committee on Immunization Practices, United States, 2020

Age group	Indication
≥2 yrs	Administration of a 2-dose MenACWY primary series in persons at increased risk for serogroups A, C, W, or Y meningococcal disease Repeated booster doses of MenACWY for certain persons who remain at increased risk for serogroups A, C, W, or Y meningococcal disease (MenACWY-D and MenACWY-CRM are licensed for a single booster dose for persons aged 15–55 yrs if at least 4 yrs have elapsed since the last dose. MenACWY-TT is licensed for a single booster dose for persons aged ≥15 yrs if at least 4 yrs have elapsed since the last dose of MenACWY)
≥10 yrs	MenB booster doses in certain persons who remain at increased risk for serogroup B meningococcal disease
≥26 yrs	MenB primary series administration in persons at increased risk for serogroup B meningococcal disease
≥56 yrs	Administration of MenACWY-D or MenACWY-CRM in persons at increased risk for serogroups A, C, W, or Y meningococcal disease

**Abbreviations:** MenACWY = quadrivalent meningococcal conjugate vaccine; MenACWY-CRM = meningococcal groups A, C, W, and Y oligosaccharide diphtheria CRM_197_ conjugate vaccine; MenACWY-D = meningococcal groups A, C, W, and Y polysaccharide diphtheria toxoid conjugate vaccine; MenACWY-TT = meningococcal groups A, C, W, and Y polysaccharide tetanus toxoid conjugate vaccine; MenB = serogroup B meningococcal vaccine.

Because of the high risk for invasive pneumococcal disease, children with functional or anatomic asplenia or HIV infection should not be vaccinated with MenACWY-D before age 2 years to avoid interference with the immune response to 13-valent pneumococcal conjugate vaccine (PCV13); MenACWY-CRM should be used in this group. If MenACWY-D is used in persons of any age with these conditions, it should not be administered until at least 4 weeks after completion of all PCV doses.

In addition, MenACWY-D should be administered either before or at the same time as DTaP to avoid interference of DTaP with the immune response to meningococcal vaccine among children at increased risk for meningococcal disease. If MenACWY-D cannot be given before or at the same time as DTaP, it should be administered 6 months after DTaP, unless the child is at increased risk for meningococcal disease because of travel to an area where disease is hyperendemic or epidemic or where an outbreak is occurring, in which case MenACWY-D should be administered regardless of timing of DTaP receipt. If MenACWY-D is inadvertently administered in the 6 months after DTaP administration, the dose does not need to be repeated.

If a healthy person aged ≥2 years previously vaccinated with a single dose of MenACWY develops an underlying condition for which meningococcal vaccination is recommended as a 2-dose primary series, a second dose should be administered as soon as possible, provided that an 8-week minimum interval between doses is maintained. For example, a person who received a single MenACWY dose before travel and then years later developed asplenia should receive another dose as soon as possible to complete the 2-dose series recommended for persons with asplenia; restarting the 2-dose series is not required. Booster doses should then be administered according to the schedule (Tables 4, 5, and 6), with the first dose administered either 3 or 5 years after completion of the primary series, depending on age. MenACWY vaccines are interchangeable; the same vaccine product is recommended, but not required, for all doses. Administration of MenACWY-D or MenACWY-CRM in persons aged ≥56 years, a 2-dose MenACWY primary series in persons aged ≥2 years at increased risk for meningococcal disease, administration of >1 booster dose, and administration of a booster dose in persons aged <15 years or at an interval of <4 years since the last dose are not licensed in the United States and are considered off-label ACIP recommendations (Table 11).

First-year college students living in residence halls should receive at least 1 dose of MenACWY within 5 years before college entry. The preferred timing of the most recent dose is on or after their 16th birthday. If only 1 dose of vaccine was administered before the 16th birthday, a booster dose should be administered before enrollment. Adolescents who received a first dose after their 16th birthday do not need another dose before college entry unless >5 years have elapsed since the dose.

#### MenB Vaccines

For persons at increased risk for meningococcal disease (Box 1), including during serogroup B meningococcal disease outbreaks, either a 3-dose MenB-FHbp series or 2-dose MenB-4C primary series should be administered. For persons who previously completed a MenB primary series who become or remain at increased risk for meningococcal disease, booster vaccination should be administered according to the dosing schedule (Tables 4, 5, 7, and 8). Primary series vaccination in persons aged ≥26 years and booster vaccination in persons at increased risk for meningococcal disease are not licensed in the United States and are considered off-label (Table 11).

For the MenB-FHbp primary series, the 3-dose series (at 0, 1–2, and 6 months) should be administered to provide earlier protection and maximize short-term immunogenicity. If the second dose is administered at an interval of ≥6 months, a third dose does not need to be administered. If the third dose is administered earlier than 4 months after the second dose, a fourth dose should be repeated at least 4 months after the third dose. For MenB-4C, doses should be administered at 0 and ≥1 months. The two MenB vaccines are not interchangeable; the same vaccine product must be used for all doses, including booster doses. Because efficacy has not been established for persons receiving MenB vaccines interchangeably, every effort should be made to determine vaccine product for all received doses, including booster doses, because receiving mismatched MenB vaccine products might result in inadequate protection (see Vaccination of Adolescents and Adults). For situations in which a dose or doses must be repeated, a minimum interval of 4 weeks should be used between any 2 doses. MenB vaccines can be administered simultaneously with other vaccines indicated for this age group, but at a different anatomic site, if feasible.

### Establishment of Vaccine-Mediated Immunity

ACIP does not recommend evaluation of antibody titers against meningococcal serogroups for the purposes of establishing immunity or the need for vaccination. Commercially available immunoglobulin (e.g., IgG) testing should not be used to infer individual seroprotection against meningococcal disease.

### Precautions and Contraindications

Because postvaccine syncope can occur with all injectable vaccines, procedures should be in place to prevent falling injuries and manage syncopal reactions. Vaccine providers, particularly when vaccinating adolescents, should consider observing patients (with patients seated or lying down) for 15 minutes after vaccination to decrease the risk for injury should they faint. If syncope develops, patients should be observed until symptoms resolve (*241*). Similarly, anaphylaxis can occur after any vaccination. Furthermore, because the tip caps of prefilled MenB-4C syringes contain natural rubber latex and might cause allergic reactions, latex sensitivity is included as a precaution for MenB-4C (*70*). Appropriate medical treatment must be available should an acute allergic reaction, including an anaphylactic reaction, occur. In addition, because apnea after intramuscular vaccination has been observed in some infants born prematurely, prematurity is a precaution for MenACWY-CRM vaccination (*66*). Finally, although postlicensure data have not established a risk for Guillain-Barré syndrome after MenACWY-D vaccination, previous history of Guillain-Barré syndrome is listed as a precaution for MenACWY-D in the package insert (*67*).

For all meningococcal vaccines, severe allergic reaction to a previous dose or any component of the vaccine is a contraindication to vaccination (*66*–*70*). For MenACWY-D and MenACWY-CRM, severe allergic reaction to any diphtheria toxoid– or CRM_197_–containing vaccine is also a contraindication (*66*,*67*). For MenACWY-TT, severe allergic reaction to a tetanus toxoid–containing vaccine is also a contraindication (*68*).

### Pregnancy and Lactation

Pregnant and lactating women should receive MenACWY vaccine if indicated. Because limited data are available for MenB vaccination during pregnancy, vaccination with MenB should be deferred unless the woman is at increased risk and, after consultation with her health care provider, the benefits of vaccination are considered to outweigh the potential risks.

### Reporting of Vaccine Adverse Events

Adverse events that occur in a patient following meningococcal vaccination can be reported to VAERS. Reporting is encouraged for any clinically significant adverse event even if it is uncertain whether the vaccine caused the event. Information on how to submit a report to VAERS is available at https://vaers.hhs.gov or by calling 1-800-822-7967.

## Future Directions in Meningococcal Vaccination

Although meningococcal disease incidence in the United States is low and decreasing, continued surveillance and evaluations are needed to assess the safety and effectiveness of MenB vaccines, including repeated booster doses among persons at increased risk for meningococcal disease. In addition to MenB, continued monitoring of MenACWY use is necessary to help evaluate the meningococcal vaccination program and provide information about the need and strategy for additional meningococcal vaccines, such as investigational serogroups A, B, C, W, and Y (MenABCWY) vaccines, in the United States (*210*,*242*). Furthermore, efforts are under way to reduce the global incidence of meningococcal disease and other causes of meningitis through a strategy that includes optimizing the use of current vaccines as well as developing additional vaccines, such as an expanded conjugate vaccine that includes serogroups A, C, W, Y, and X for use in sub-Saharan Africa (*243*,*244*).
